# MgO–water interface: structure and surface dissolution depend on flow and pH

**DOI:** 10.1039/d5cp03295d

**Published:** 2025-10-10

**Authors:** Moritz Zelenka, Ellen H. G. Backus

**Affiliations:** a University of Vienna, Faculty of Chemistry, Institute of Physical Chemistry Währinger Straße 42 1090 Vienna Austria ellen.backus@univie.ac.at; b University of Vienna, Vienna Doctoral School in Chemistry (DoSChem) Währinger Straße 42 1090 Vienna Austria

## Abstract

Magnesium oxide (MgO) is frequently in contact with water throughout numerous research and industrial applications and in nature. Remarkably, we found that there is a substantial influence on the interfacial structure and dissolution process whether water is flowing or static at the MgO(100) surface. Sum frequency generation spectroscopy revealed that flowing acidic solutions enhance the charging of the MgO surface, which leads to an increased net orientation of water close to the surface. Contrary, the MgO surface resembles a near neutrally charged state when in contact with static liquid for all tested solutions between pH 3 and pH 11. We explain this surprising observation with the dissolution of MgO in aqueous solutions, which effectively removes charge from the interfacial region. The continuous solution exchange due to flowing liquid shifts the equilibrium towards a more charged state in comparison to static liquid. Additionally, by investigating the transition from flowing to static liquid we found a reaction order of around 0.5 for the dissolution reaction with respect to the H^+^ concentration. Furthermore, the significant effect of the MgO surface dissolution on the interfacial structure points out that other solid–liquid interfaces with similar or higher solubility might exhibit similar properties.

## Abstract 2

The interplay between the molecular structure of liquid water and the dissolution reaction at the mineral magnesium oxide (MgO) is revealed by sum frequency generation spectroscopy with alternating flowing and static liquid phase.

## Graphical abstract

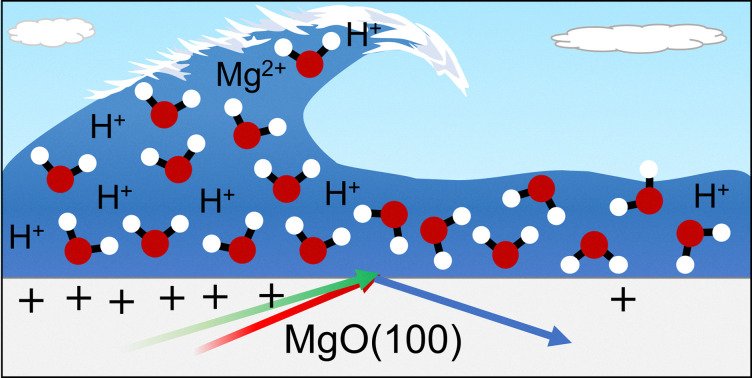

## Introduction

Understanding the interplay of oxide surfaces, such as magnesium oxide (MgO), with water is crucial for a plethora of research areas and industrial applications, such as heterogenous catalysis,^1^ construction,^2^ and nanoscience.^3^ Furthermore, as both oxides and water are commonly in contact with each other in nature, their interactions have a significant impact on geological and atmospheric processes affecting our environment and climate.^4^ However, studying and understanding these oxide–water interactions down to the molecular level often is far from trivial as oxide surfaces can be very dynamic and reconstruct or dissolve when in contact with water.^5,6^ As a result, even for MgO, a moderately simple oxide, the interfacial structure and surface dissolution process are despite substantial research efforts not yet fully understood. It is well researched both experimentally and theoretically that the MgO surface gets hydroxylated when in contact with gaseous or liquid water, with additional adsorbed water molecules present at the interface.^7–13^ Further, it has been shown that altering the aqueous phase pH directly affects the distribution of these interfacial species and changes the resulting surface charge by interactions with positively charged H^+^ or negatively charged OH^−^ ions.^14–17^ Yet, it is not known if and how the MgO dissolution affects the interfacial water structure and surface charge, especially in acidic solutions where the dissolution rate is the highest. The effect on the MgO surface itself was studied by atomic force microscopy.^18^ It was found that during the dissolution holes in the low μm range were formed on the MgO(100) surface and that the (110) and (111) surfaces seemed to dissolve such that the most stable (100) facet was formed. Furthermore, the rate of hole formation upon flowing HCl solutions along the MgO surface was correlated with the dissolution reaction to obtain reaction rates. It was concluded that the dissolution was surface controlled rather than by H^+^ diffusion from the bulk and changed only little between pH 1–2.^18^ Mostly throughout literature the dissolution reaction was not studied at the surface but rather in bulk by transferring a known amount of solid MgO in an (acidic) aqueous solution and following the increase of the pH, *i.e.*, the decrease of the H^+^ concentration, as a function of time. The change in H^+^ concentration is linked to the dissolution according to the following net reaction, where ≡MgO (s) refers to the MgO surface:

1 ≡MgO (s) + 2H^+^ (aq) → Mg^2+^ (aq) + H_2_O (aq)

Within this approach it is assumed that a bulk pH measurement can be linked to the surface reaction when exchanging the aqueous solution at the MgO surface fast enough, for example by stirring.^19,20^ Generally, it was observed that the reaction rate was proportional to the H^+^ concentration of the solution.^20–23^ H^+^ adsorption to the surface and the following desorption of dissolution products was found to be determining the rate below pH 5. Above pH 5 diffusion limitation occurred for H^+^, while above pH 7 OH^−^ adsorption was discussed as rate determining.^20,23^ Identifying an individual slowest elementary reaction step that controls the reaction rate has proven difficult, as multiple equilibria and reaction steps contribute to the dissolution. A useful parameter for such investigations is the reaction order, which relates the observed rate to the concentration of reactants. Throughout literature different reaction orders with respect to the H^+^ concentration were calculated and values around 0.5^19–23^ are most common to our knowledge. However, several different rate limiting reaction steps can explain a reaction order of 0.5, such as a second protonation step^21,22^ or dissolution of Mg^2+^ from the surface.^19^

In this article we want to bridge the gap between studies on the interfacial water structure and the surface dissolution and investigate how they influence one another. To that goal we also must consider an underlying property found to the best of our knowledge for all experiments throughout literature, namely that in dissolution experiments the liquid at the MgO surface was moving by an external force like stirring,^18–23^ while in water structure studies the liquid was static.^7–16^ This might seem insignificant on a first glance, but it was shown for SiO_2_^24,25^ and CaF_2_^24,26,27^ that motion of the liquid can alter the interfacial chemistry and surface charge significantly. Thus, as a first step we investigate the MgO–H_2_O interface using flowing and static liquid. We probe aqueous phases between pH 3 and 11 with a constant ionic strength of 1 mM, because it was found that both the surface charge and the dissolution are pH dependent. To obtain molecular level insights into the system we employ sum frequency generation (SFG) spectroscopy. This technique is reporting specifically on vibrations of interfacial species, also in the presence of centrosymmetric bulk phases such as crystalline MgO or water.^28,29^ The inherent interface selectivity of this technique allows us to perform the measurements using macroscopic amounts of water at room temperature and ambient pressure.

## Results and discussion

### MgO–H_2_O interfacial structure with flowing and static liquid

**Fig. 1:**
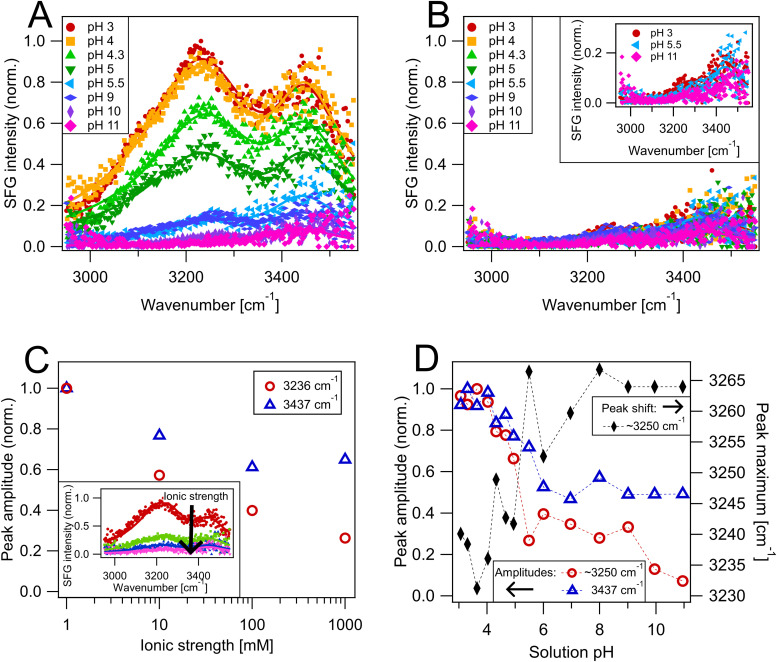
Steady state SFG spectra of the MgO(100)–H_2_O interface with different aqueous solutions. The spectra are normalized to the maximum SFG intensity of the MgO–H_2_O interface at pH 3 with flowing liquid. Measurements with aqueous solutions between pH 3 and 11 and a constant ionic strength of 1 mM are depicted for (A) flowing and (B) static liquid. Solid lines are fits to the data. The inset in (B) shows selected spectra with an enlarged *y*-axis scale. (C) Surface charge screening experiments using flowing liquid at pH 3 with ionic strengths ranging from 1 mM to 1000 mM adjusted by adding NaCl. The normalized amplitudes of the 3236 cm^−1^ and 3437 cm^−1^ peaks are obtained by fitting the SFG spectra shown in the inset. (D) Plot of the peak amplitudes *versus* the aqueous phase pH value obtained from fitting the spectra under liquid flow shown in (A). Additionally, the peak shift of the ∼3240 cm^−1^ band is shown on the right axis. The data points are connected by lines to guide the eye.

SFG spectroscopy was employed to investigate the interface of MgO–H_2_O with both flowing and static liquid at the MgO(100) surface. The spectra were recorded in the O–H stretching mode region (3000–3550 cm^−1^), allowing to investigate the nature, orientation and hydrogen bonding environment of water molecules. Measurements with flowing liquid along the MgO surface are depicted in Fig. 1A. The probed aqueous solutions had pH values between pH 3 and 11, adjusted with HCl and NaOH. Furthermore, the ionic strength of the solutions was kept constant at 1 mM since different ionic strengths can alter the measured SFG intensity.^30^ The spectra in Fig. 1A show a clear dependence of the measured SFG intensity on the solution pH value. From pH 3 to pH 4 there was a plateau where the measured SFG intensity was the highest. A further increase of the solution pH, *i.e.*, a reduction of the H^+^ concentration, caused the SFG intensity to decrease significantly, which is observable in the measurements with pH 4.3, pH 5 and pH 5.5 solutions. Subsequent measurements with pH 6 to pH 11 solutions showed a further moderate decrease of the SFG intensity. Despite the drastic changes in intensity, the spectral shape did not change. There were always two roughly equally intense peaks across all measured pH solutions. One peak was centred around 3240 cm^−1^ and the other one was centred around 3440 cm^−1^. Surprisingly, drastic differences were obtained when the liquid remained static in the measurement cell instead of flowing. For static liquid the solution exchange in the interfacial region was only driven by diffusion processes. SFG spectra with static liquid are shown in Fig. 1B. Overall, the spectra still showed two peaks at ∼3240 cm^−1^ and ∼3440 cm^−1^ similar to the case of flowing liquid (Fig. 1A), but the measured intensities were significantly lower. Additionally, the SFG intensity was not markedly affected by an alteration of the pH value of the liquid phase. All recorded static liquid spectra from pH 3 to pH 11 had comparable SFG intensities to the spectra measured with flowing liquid at pH 10 or pH 11. This unexpected strong difference of the signal intensity between flowing and static liquid at MgO means that the interfacial region must be altered markedly. The observation of two peaks with similar peak frequencies in both cases, indicates that the interfacial species causing these peaks are the same for flowing and static liquid. In SFG spectroscopy the measured intensity is proportional to the net polarization at the interface.^29^ Such polarization effects can result from a net orientation of water molecules caused by interactions with the MgO surface. Yet, the strong influence of the aqueous phase pH value on the recorded SFG intensity when using flowing liquid, as well as the pronounced intensity decrease in case of a static liquid, suggests an additional source of polarization – a surface charge.^31^ As a basic oxide, the MgO surface is positively charged by adsorbing H^+^ up to pH 10–12.^15–17,32^ The resulting electric potential exerts a force on the water molecules within the liquid medium near the MgO surface, which affects their charge distribution and orientation. Depending on the solution properties, such as the ionic strength, the orienting/polarising effect of the surface electric field is not limited only to the first interfacial layers. In this case the SFG signal would also have contributions from water molecules further away from the MgO surface,^30,31,33^ which we hereafter will refer to as diffuse layer contribution, as defined in the Stern double layer model.^34^

To experimentally test if a charged MgO interface influences the observed pH sensitive and flow on/off behaviour, we conducted experiments on the MgO–H_2_O interface at pH 3 with flowing liquid where the ionic strength of the used solutions was successively increased from 1–1000 mM by adding NaCl. We fitted the data with two peaks centred at 3236 cm^−1^ and 3437 cm^−1^ to better quantify the spectral changes. The remaining fitting parameters are summarised in Table S3. The resulting peak amplitudes are depicted in the main graph in Fig. 1C, while the inset shows the full spectra. For the peak at 3236 cm^−1^ an amplitude reduction of around 40% was observed when increasing the ionic strength from 1 mM to 10 mM. A further elevation of the ionic strength to 1000 mM caused the peak amplitude to decrease by about 75% compared to the 1 mM measurement. This behaviour validates the hypothesis of a charged MgO surface contributing to the measured signal^30,33^ and can be explained as follows: ions in solution are able to screen the surface electric field *via* double layer formation. The higher the ionic strength the more the surface field is screened, which reduces its penetration depth into the liquid phase and followingly also reduces the thickness of the diffuse layer. Thus, by increasing the ionic strength the number of net oriented/polarised water molecules is decreased which in turn decreases the measured SFG intensity. The very strong influence of the ionic strength on the amplitude of the 3236 cm^−1^ peak suggests that it is predominantly originating from water molecules from the diffuse layer that only feature net orientation or polarization because of a charged MgO surface.^30^ These water molecules have strong hydrogen bond interactions, which explains the low O–H stretch frequency comparable to bulk water.^35^ The amplitude of the second peak at 3437 cm^−1^ also decreased with increasing ionic strengths as depicted in Fig. 1C, although the reduction was distinctively weaker compared to the 3236 cm^−1^ peak. When the ionic strength was increased from 1 mM to 10 mM the peak amplitude decreased only by about 25%, half of what was observed for the 3236 cm^−1^ peak. Furthermore, the 3437 cm^−1^ peak amplitude decreased only by around 40% when the ionic strength was increased to 100 mM. No additional significant reduction was observable at 1000 mM. It was reported that the diffuse layer water has a spectrum with features around 3200 cm^−1^ and around 3400 cm^−1^,^34,36^ which explains the decreasing amplitude of the 3437 cm^−1^ peak when the surface charge is more screened. Still, the significantly smaller decrease of the 3437 cm^−1^ peak amplitude compared to the 3236 cm^−1^ peak implies that there is an additional effect contributing to the net orientation of this species that cannot be explained by the surface charge alone. We thus assign this peak partly to adsorbed H_2_O molecules, which show a net orientation due to binding to the MgO surface. Moreover, the higher frequency of this peak signifies that the adsorbed water has a weaker hydrogen bond network than the diffuse layer water.^37^ The presence of surface adsorbed H_2_O was also proposed by earlier work both on neutral surfaces and across the entire pH range with similar vibrational frequencies.^7,11,15,16^ A more detailed investigation of the vibrational peaks is given in the SI in Section S3 using conventional SFG measurements with a broader wavenumber range, as well as phase-resolved spectra, of MgO–H_2_O at pH 3. These measurements imply that the 3437 cm^−1^ feature is actually a combination of two different peaks, most likely from adsorbed water at different binding sites. Furthermore, we found vibrational peaks above 3550 cm^−1^, which are assigned to hydroxyls at the MgO surface in accordance with literature.^7,8,38^ It was also deduced from the phase-resolved measurements that all found species, diffuse layer water, adsorbed water and hydroxyls point on average with the oxygen atom towards the MgO surface.

The pronounced influence of the surface charge on the MgO–H_2_O spectra suggests that it plays a role in the striking differences observed for flowing *versus* static liquid as well. The MgO surface is positively charged by H^+^ adsorption, therefore the surface charge is dependent on the solution H^+^ concentration.^17^ Consequently, we focus again on the measurements with different pH solutions, depicted in Fig. 1A for flowing liquid and 1B for static liquid. The spectra were fitted to quantify the amplitude changes. For flowing liquid, it was found that the peak maximum of the diffuse layer water shifted as a function of the solution pH value from 3240 cm^−1^ at pH 3 to 3264 cm^−1^ above pH 9. On the contrary, the adsorbed water maximum was constant at 3437 cm^−1^. The fitted amplitudes as a function of the solution pH are depicted in Fig. 1D, while the complete fit parameters are summarised in the SI in Tables S1 and S2. Both the diffuse and adsorbed water had near constant amplitudes when solutions equal or lower than pH 4 were measured, consistent with the spectra in Fig. 1A. As the diffuse layer water is only influenced by the charge at the MgO surface, a constant amplitude indicates a saturation of the charge when the H^+^ concentration is high enough. This agrees with pH dependent simulations of MgO–H_2_O, where it was found that at a certain H^+^ concentration threshold the available surface binding sites were saturated.^14,15^ After pH 4 the amplitude of the diffuse layer water contribution diminished drastically and reached an amplitude close to zero at pH 11. This decrease is directly linked to a decrease of the MgO surface charge since the ionic strength was constant. A near zero amplitude of this band observed at pH 11 indicates therefore a neutral surface, which matches literature values where the point of zero charge for MgO was found in the range of pH 10–12.^15,17,32^ To rule out counteracting effects and confirm the lack of diffuse layer polarisation at high pH values, we performed measurements of MgO–H_2_O at pH 11 with ionic strengths ranging from 1–1000 mM, which lead as expected to no effect on the measured SFG intensity (see Fig. S2). The decreasing surface charge at high pH solutions also can explains the observed shift of the diffuse layer peak to higher wavenumbers depicted in Fig. 1D. The small shift towards higher wavenumbers denotes a decreasing hydrogen bonding strength when the surface charge decreases, which matches observations for charged lipids at the water–air interface where it was found that strong electric fields enhance the hydrogen bonding strength.^39^ On the other hand, the peak maximum of the adsorbed water at 3437 cm^−1^ stayed constant, signalising that its environment is independent of the surface charge. The amplitude of the adsorbed water, depicted in Fig. 1D, showed a comparatively smaller decrease when rising the solution pH value above pH 4. At pH 11 the amplitude only decreased by about 50% compared to the plateau between pH 3 to pH 4. This means that the adsorbed water still has a net orientation even at a neutral MgO surface at pH 11.

Generally, there is a striking resemblance of the spectra with flowing liquid at pH 10 and 11 from Fig. 1A and the spectra under static liquid across the entire pH range from Fig. 1B. Accordingly, the diffuse layer peak maximum with static liquid at 3268 cm^−1^ resembles the frequency found at pH 9–11 during liquid flow. Adsorbed water was fitted again with the same frequency found for flowing liquid at 3437 cm^−1^. The inset in Fig. 1B and fitting results from Table S2 showcase that there was a small decreasing trend of the peak amplitudes with increasing pH value, especially at pH 11, which was also reported in literature.^16^ Yet, the overall similarity of the spectra implies that the MgO surface approaches a neutrally charged state in contact with a static liquid regardless of the solution pH value. This suggests that there is a mechanism that efficiently removes charge from the MgO surface. A direct influence of the liquid motion and different flow rates on the water orientation was found to be negligible for the used experimental conditions and is discussed in detail in the SI in Section S6.

### The effect of MgO dissolution on the surface charge

**Fig. 2:**
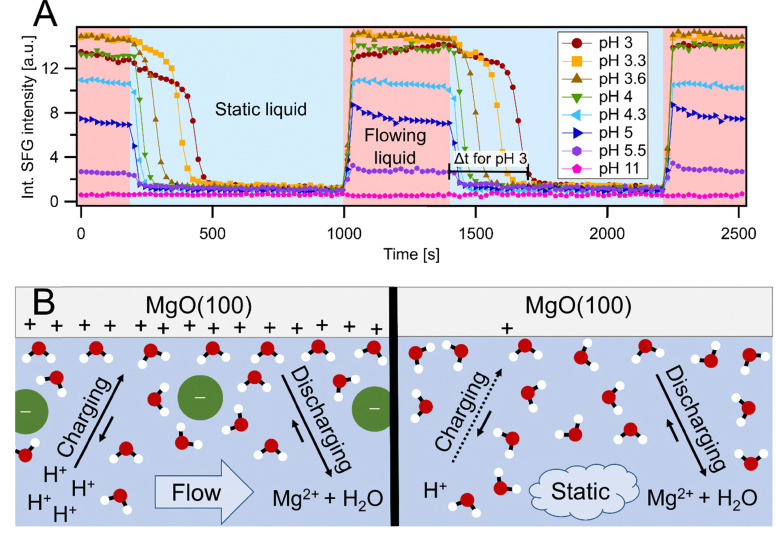
(A) Continuous SFG spectroscopy measurements of the MgO–H_2_O interface at different pH values during flowing (red area) and static (blue area) liquid cycles. Each data point corresponds to the integrated SFG intensity from 3000–3550 cm^−1^ of a corresponding 20 s spectrum. The time since the start of the experiment is displayed on the *x*-axis. Selected measurements are shown between pH 3 and 11. Data points are connected by lines to guide the eye. The intensity decay time used in eqn (2) is sketched for the second cycle. (B) Illustration of the MgO–H_2_O interface under acidic conditions, including charging by H^+^ adsorption on the MgO surface and the discharging by surface dissolution and diffusion of Mg^2+^ away from the surface. Left: Flowing liquid. Right: Static liquid.

Surface dissolution processes often contain complicated sequences of chemical equilibria and elementary reaction steps. To describe the distinct difference between liquid flow and static liquid we break down the dissolution process between pH 3 and 11 into two main parts, which encompass the net reaction given by eqn (1). First, the MgO surface gets positively charged by adsorption of H^+^ ions. Second, two H^+^ are consumed and Mg^2+^ and H_2_O are formed and diffuse from the interface to the bulk, which removes interfacial charge. The balance of these two reactions controls therefore the net charge found on the MgO surface. Our results showed that liquid motion can shift this balance towards a more charged state especially in low pH solutions, whereas the MgO surface in contact with static liquid was dominated by the discharging reaction. To test if these processes are reversible, we performed measurements in which the liquid flow was cyclically turned on and off while continuously recording spectra with 20 s acquisition time. These measurements are displayed in Fig. 2A, where the integrated intensity of each spectrum is plotted *versus* the elapsed experiment time. In the red shaded area liquid was flowing along the surface, while in the blue shaded area the flow was switched off. It is observable that upon stopping liquid motion the SFG intensity and therefore the surface charge decreased. The reduction was slower when solutions with lower pH were used. When the flow was activated again the intensities increased within seconds back to the respective maximum intensity for all tested pH solutions. Here it shall be noted that the spectra depicted in Fig. 1 are all from steady states and not from the transition phase between flowing to static liquid or *vice versa*. The reversibility of the charging and discharging, as well as the dependence on solution pH, suggests that it is the H^+^ diffusion from the bulk to the MgO surface and the surprisingly fast discharging reaction that cause the distinct flow on/off behaviour.

Fig. 2B shows a sketch of the proposed dynamics at the interface. On the left side the interface under liquid flow is displayed. The main characteristic of a flowing liquid is that the solution in the interfacial region is steadily exchanged. As such, it can be approximated to be identical with the bulk liquid. This assumption is based on experiments which showed that varying the flow speed does not influence the recorded spectra in the limit of moderate laminar flow (SI Section S6) and on calculations of the ion diffusion *versus* the fluid velocity (see SI, Section S5). The observed constant intensity between pH 3 and 4 indicates that the H^+^ concentration is high enough to saturate the MgO binding sites and the dissolution is likely rate determined by the removal of Mg^2+^ and H_2_O from the surface. Above pH 4 the dynamics change, noticeable by the lower SFG intensities. Consequently, above pH 4 the charging of the surface becomes more sluggish due to the lower H^+^ concentration in the solution and as the pH increases the discharging becomes more and more dominant, since it is less dependent on the solution pH.

Similar reasoning can be applied for a static liquid phase, depicted in Fig. 2B on the right. There is still a balance of the charging and discharging reactions. However, for a static liquid phase the solution at the interface is not readily exchanged anymore. As a result, the H^+^ concentration of the interfacial region depletes over time because H^+^ is consumed in the dissolution reaction, which can only be compensated by diffusion from the bulk in a static solution. As the observed SFG intensities were not significantly varying across the measured pH range (see Fig. 1B), it follows that the MgO surface charge is effectively diminished irrespective of the bulk pH. This implies two things. First, the surface dissolution reaction is fast enough to drain the H^+^ concentration of the interfacial region. Second, Mg^2+^ is also under static conditions effectively removed from the interface by diffusion, since otherwise a positive interfacial charge would be detected. We performed additional experiments using pH 3 solutions with 1–100 mM MgCl_2_ added to further challenge the hypothesis that Mg^2+^ has no impact on the dissolution process and surface charge. The measurements are presented in the SI in Section S7, where it was found that Mg^2+^ indeed has no effect in the low concentration limit relevant for the solutions and conditions used within this study. Just at artificially high concentrations of ≥10 mM MgCl_2_ an acceleration of the charge decrease was found. These findings are supported by kinetic studies, where it was found as well that the addition of Mg^2+^ ions to the measurement solution did not have a significant effect on the dissolution rate, even at Mg^2+^ : H^+^ ratios of 10 : 1.^18,21^

### Liquid flow on/off transition and the reaction order

The observed dynamics upon switching off the liquid flow can be used to draw conclusions about the reaction order and rate determining step of the dissolution. It was already concluded that the flow on/off dynamics are primarily driven by a H^+^ concentration depletion in the interfacial region, which is in line with a slower relative intensity decrease at low pH (Fig. 2A). A pH 3 solution has a 10 times higher H^+^ concentration than a pH 4 solution and therefore it takes longer until the interfacial H^+^ concentration reaches the threshold where diffusion of H^+^ to the surface becomes slower than the following surface dissolution. It should be pointed out, that the diffusion could be artificially slowed down by the geometry of the measurement cell. Thus, we investigated the effect of the measurement cell in Section S5 of the SI and inferred that possible contributions from it can be neglected. The depletion of the H^+^ concentration near the MgO surface after liquid motion is stopped, can be used to calculate the reaction order of the acidic MgO dissolution similar to studies measuring the reaction by bulk properties.^19–23^ The reaction rate *r* of the dissolution can be expressed by2
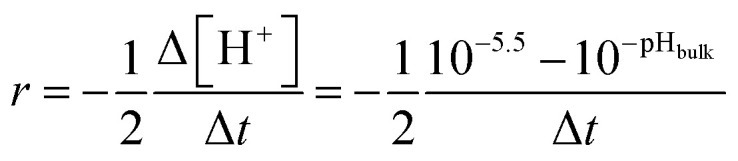
where Δ[H^+^] is the H^+^ concentration change at the interface after switching off liquid motion and Δ*t* corresponds to the respective time span of the concentration change, as sketched in Fig. 2A. The ½ accounts for the net reaction stoichiometry from eqn (1), as two H^+^ are consumed during the dissolution reaction. Δ[H^+^] can be obtained as follows: the starting interfacial H^+^ concentration, *i.e.*, the concentration directly when the flow is switched off, is approximated by the bulk pH, pH_bulk_. The chosen end H^+^ concentration is reached when the measured intensity and spectrum match the measurement of a pH 5.5 solution under liquid flow, which in turn indicates an interfacial H^+^ concentration of pH 5.5. Please note that using another end concentration or final point does not significantly change our results, as long as the reference solution is not in the charge saturation regime below pH 4. It is not possible to compare the calculated reaction rate with literature values, since we cannot normalise for the probed surface area using our approach. Still, a simple rate law for the reaction based on the calculated rates *r* can be expressed as follows:

3 *r* = *k*[H^+^]^*n*^

where *n* is the reaction order and [H^+^] the starting H^+^ concentration identical to the bulk pH value. The dependence on the MgO concentration is included in the rate constant *k*, as it is a solid and thus not considered to undergo concentration changes. Eqn (3) can be linearized and expressed in terms of pH instead of [H^+^] by taking the decimal logarithm:

4 log_10_(*r*) = log_10_(*k*) − *n*·pH_bulk_

**Fig. 3:**
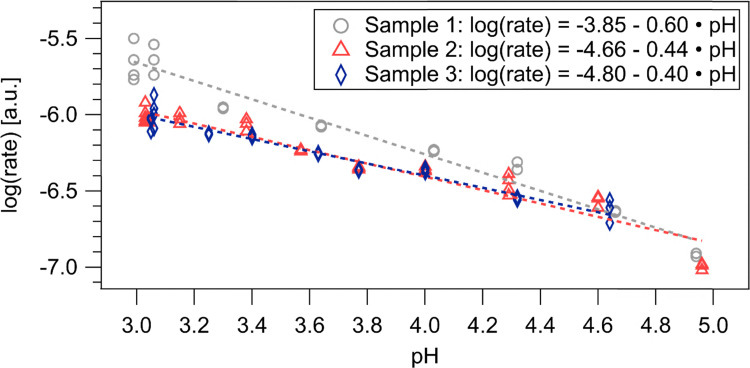
Calculated log(rate) *versus* initial/bulk pH obtained from the pH dependent SFG intensity decrease after switching of the flow (see Fig. 2A). The data is fitted with a line according to eqn (4). The different symbols and colours correspond to three different MgO substrates.

It shall be noted that the integral method to evaluate the reaction order *n* is less applicable in our case than the initial rates approach, since we are measuring an open system, where the interface is connected to a reservoir whose pH is not considered to change. The measured log_10_(*r*) *vs.* pH_bulk_ data points are depicted in Fig. 3. There is a notable spread in the data, likely from influences by slight surface roughening during dissolution and general fluctuations between different samples and measurement days which is discussed in the SI in Section S8. To account for the variability, we measured in total 85 data points on three different MgO substrates. The data of each sample was fitted individually with eqn (4) to specifically account for the effect of different samples. The average of the slopes *n̄* and the corresponding standard deviation s were calculated to yield a reaction order of 0.48 ± 0.11 (*n̄* ± *s*). Please note that in our experimental approach we cannot access small concentration changes needed for true initial rates, as the SFG intensity is saturated below pH 4 (Fig. 1A and 2A). Additionally, the near constant SFG signal after pH 5.5 (Fig. 1A) prohibits us from taking identical time intervals for the initial rates. As best approximation, we averaged the rate over variable time periods as described in eqn (2). Model calculations with various kinetic rate laws showed that this leads to an underestimation of the reaction order. Nevertheless, several studies reported values for the reaction order around 0.5,^19,21–23^ which matches our result within the determined uncertainty. Yet, throughout literature an ambiguity in the assignment of an appropriate rate determining step arose, because multiple models fitting a reaction order of 0.5 were suggested.^21^ Based on our interface specific experiments we suggest that this uncertainty results from a delicate pH dependence of the rate determining step. When diffusion control of the reaction is counteracted by flowing liquid the saturated surface charge below pH 4 suggests that the surface protonation is fast and the dissolution of Mg^2+^ gets rate determining, in line with the argumentation of ref. 19. Above pH 4 the reduced surface charge indicates that H^+^ adsorption is becoming more sluggish and becomes rate determining, which was suggested as the rate determining step in ref. 21 and 22. A pH dependence of the rate determining step is also indicated by the initial slow and then fast SFG intensity decrease after stopping liquid flow observed in Fig. 2A for solutions with pH < 4. We did observe negligible surface charging under steady state static liquid conditions between pH 3 and 11, suggesting that the surface dissolution is generally faster than H^+^ diffusion to the surface, which therefore is rate determining in static liquid. This was surprising to us, since the H^+^ diffusion is considerably fast in aqueous solutions and motivates therefore future research into the time dependent ion distributions of MgO in contact with acidic aqueous solutions.

## Conclusion

The interfacial structure of MgO in contact with water was studied by sum frequency generation spectroscopy in the O–H stretch region (2800–3800 cm^−1^). Next to hydroxyls above 3500 cm^−1^, we observed two different species: water molecules in the diffuse layer, *i.e.*, beyond the first few interfacial layers, around 3240 cm^−1^ and adsorbed water molecules around 3440 cm^−1^. A substantial dependence of the net orientation and polarisation of these peaks on the MgO surface charge was observed, which in turn was influenced by the liquid phase pH and whether the liquid was flowing or remained static. The surface charge was constant for flowing liquid between pH 3 and 4, which indicates that the MgO surface was saturated with adsorbed H^+^. At increasing pH, the surface charge decreased until a neutral state was reached around pH 11. Surprisingly, a near neutral surface was observed when the liquid remained static below the MgO surface, irrespective of the solution pH value. The drastic difference is rationalised by the dissolution of MgO in aqueous solutions, which depletes the interfacial H^+^ concentration and discharges the MgO surface. With flowing liquid the H^+^ concentration depletion is counteracted by the fluid exchange, whereas under static liquid only diffusion from the bulk is compensating for the H^+^ concentration reduction. From the lack of surface charge at static liquid, we conclude that the surface dissolution is fast enough to deplete the interfacial H^+^ concentration. Based on the transition from flowing to static liquid, we calculated a reaction order of 0.48 ± 0.11 for the dissolution reaction with respect to the H^+^ concentration. Furthermore, we propose that the rate determining step is highly dependent on the solution pH, which might explain the controversy in literature.

Generally, this article demonstrates that solid–liquid interface can be decisively altered by dynamic phenomena and chemical reactions. The observed effects were markedly stronger than earlier findings in literature on CaF_2_ and SiO_2_, which are both more stable than MgO under the tested conditions. As such similar findings might be made for materials with similar or higher dissolution than MgO, such as other oxides like CaCO_3_ under acidic conditions or metals in oxidising environments.

## Author contributions

Moritz Zelenka: conceptualisation, methodology, investigation, validation, formal analysis, writing – original draft; Ellen H. G. Backus: conceptualisation, methodology, validation, resources, writing – review & editing, supervision, funding acquisition.

## Conflicts of interest

There are no conflicts to declare.

## Supplementary Material

CP-027-D5CP03295D-s001

## Data Availability

Experimental details and experiments supporting conclusions from the main text can be found in the supplementary information (SI). The authors have cited additional references within the SI.^40–48^ See DOI: https://doi.org/10.1039/d5cp03295d. Data for this article are available at the PHAIDRA repository of the University of Vienna at https://phaidra.univie.ac.at/o:2169457.
