# Supplementary material for: MgO–water interface: structure and surface dissolution depend on flow and pH
Source: Phys Chem Chem Phys. 2025 Oct 10;27(43):23356–64. doi: 10.1039/d5cp03295d (PMC12539936; doi:10.1039/d5cp03295d)
Supplement: CP-027-D5CP03295D-s001 [file CP-027-D5CP03295D-s001.pdf]

Supplementary Information (SI) for Physical Chemistry Chemical Physics.
This journal is © the Owner Societies 2025

Supporting information: “MgO-water interface: Structure and surface dissolution depend on flow and pH”

Moritz Zelenka[1,2] and Ellen H. G. Backus*[1,2]

[1] University of Vienna, Faculty of Chemistry, Institute of Physical Chemistry, Währinger Str. 42, 1090 Vienna, Austria.

[2] University of Vienna, Vienna Doctoral School in Chemistry (DoSChem), Währinger Str. 42, 1090 Vienna, Austria.

*Corresponding author: ellen.backus@univie.ac.at

Contents

S1: Experimental Section 2

Materials 2

Measurement cell and calculation of Reynolds number 2

Conventional sum frequency generation spectroscopy 3

Phase-resolved sum frequency generation spectroscopy 4

Fitting of conventional sum frequency generation spectra 5

S2: Surface charge screening experiment at pH 11 7

S3: Conventional and phase-resolved sum frequency generation spectroscopy measurements using higher resolution 8

S4: Transmission infrared spectroscopy of the MgO sample 11

S5: Probing the influence of the measurement cell on the flow on/off dynamics 13

S6: The effect of different fluid flow rates on the observed spectrum 15

S7: Effect of Mg^{2+} addition on the signal intensity and flow on/off transition 17

S8: Effect of dissolution on the MgO surface 19

References 20

S1: Experimental Section

Materials

Optically polished MgO crystals (25.4 mm diameter, 2 mm thickness) with (100) surface orientation[1] were purchased from Crystal GmbH. Before measurements the substrates were heated at 500°C for 2 h to remove contaminations. Aqueous solutions in the range of pH 3 to 11 were prepared using HCl (>37%, CHEMSOLUTE), NaOH (>99.5%, CHEMSOLUTE) and ultrapure Water (Milli-Q, Merck). Generally, an ionic strength of 1 mM was maintained throughout the measurements. Solutions of higher ionic strength were made using NaCl (≥99%, ACS reagent, Sigma-Aldrich) and $MgCl_2$ (anhydrous, Sigma). The pH of the solutions was measured using a pH meter (FiveEasy Plus, METTLER TOLEDO). All experiments were carried out at ambient pressure in a temperature-controlled lab at 20°C. All measurement solutions were equilibrated to 20°C as well.

Measurement cell and calculation of Reynolds number

Experiments with liquid flow, unless otherwise noted, were conducted with volumetric flow rates of 6 $mL{\cdot}min^{-1}$ using a peristaltic pump (Masterflex L/S, Cole-Parmer) and Tygon 2011 tubing (Cole-Parmer) attached to the measurement/flow cell. Drawings of the cell can be found in Figure S1. For static liquid measurements the pump was switched off. Solutions were not circulated but disposed after passing through the cell.

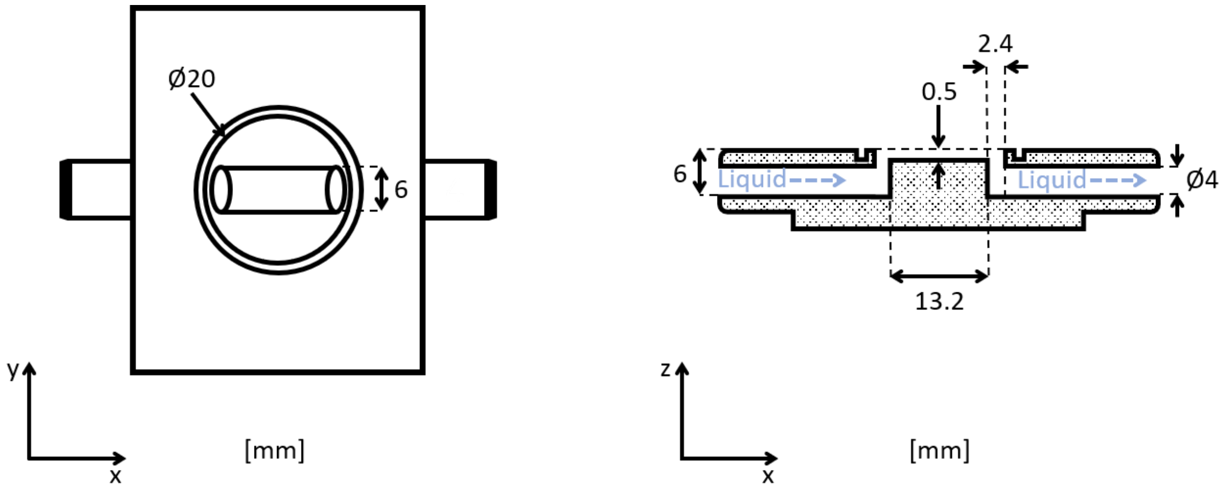

Figure S1. *Drawings of the stainless-steel flow cell. Measures are in mm. On the left a top view of the cell is depicted, while on the righthand side a side view, also indicating the liquid flow, is sketched. The MgO discs were mounted on top of the cell and sealed using an FKM sealing ring, thus the effective channel height was ca. 1 mm on top of the channel and ca. 0.5 mm on the side of the channel. Measurements were always conducted at the centre of the channel, unless otherwise noted.*

[1] For cubic MgO crystals the planes (100), (010) and (001) are equivalent.

The fluid flow through the channel was supposed to be laminar within this study. Whether a current is laminar or turbulent can be estimated using the Reynolds number, which is defined by the following equation [40]:

$$Re = \frac{\rho \cdot u \cdot L}{\mu} \qquad (1)$$

where ρ is the density of the liquid and μ is the dynamic viscosity. L is the characteristic length, which is approximated for the used channel by the hydraulic diameter of a rectangular duct, which yields the following expression [40]:

$$L = \frac{2 \cdot a \cdot b}{a+b} \qquad (2)$$

In this equation a and b are the width and the height of the rectangular channel. For the flow cell used in our experiments the channel width was 6 mm and the height 0.5 mm, which results in a characteristic length of 0.001 m. The fluid velocity u is derived by dividing the volumetric flow Q through the cell by the channel area A:

$$u = \frac{Q}{A} \qquad (3)$$

A volumetric flow of 6 $mL \cdot min^{-1}$ (=10^{-7} $m^3 \cdot s^{-1}$) and a channel area of $3 \cdot 10^{-6}$ m^2 results in a fluid velocity of 0.03 m/s. The remaining parameters can be approximated by the values of pure water, which has a dynamic viscosity of $1.002 \cdot 10^{-3}$ $kg \cdot m^{-1} \cdot s^{-1}$ and a density of 998 $kg \cdot m^{-3}$ at 20°C [41]. The resulting Reynolds number is 31, which is well below the usual limit for turbulent flow [40]. During measurements the substrate was placed on top of a FKM O-ring to seal the cell, which elevated the substrate. Consequently, the actual depth of the liquid was determined to be around 1 mm below the channel and around 0.5 mm next to the channel using a distance sensor (CL-L070, Keyence). If a channel depth of 1 mm is assumed the calculated Reynolds number is 7% lower.

Conventional sum frequency generation spectroscopy

Sum frequency generation spectroscopy was performed using a Ti:sapphire laser (Libra, Coherent). The 800 nm pulses had a duration of around 40 fs with a 1 kHz repetition rate. A part of the 800 nm output was sent through an etalon to yield pulses with a reduced bandwidth of about 20 cm^{-1} full width at half maximum (FWHM) and about 20 µJ power. Another part of the laser output was used to generate the infrared pulses using an optical parametric amplifier (TOPAS PRIME, LIGHT CONVERSION) with consecutive non-collinear difference frequency generation (NDFG, LIGHT CONVERSION). The produced infrared pulses had a bandwidth of about 300 cm^{-1} FWHM at 3100 nm and between 4-5 µJ power. The 800 nm and infrared pulses were focused onto the probed interface at about 60° and 40° angle of incidence with respect to the surface normal, respectively. For all measurements the MgO substrates were mounted on a stainless-steel flow cell described above, where the infrared and 800 nm laser pulses were passed through the MgO substrate first before reaching the MgO-H_2O interface and the reflected sum frequency light from the interface was collected. The produced sum frequency light was first dispersed using a 600 g/mm grating (Andor Shamrock 303i, Oxford Instruments) and detected with a charge-coupled device image sensor (Andor Newton EMCCD, Oxford Instruments), allowing to bin selectively over the signal from the MgO-water interface excluding any potential signal from the MgO-air interface. With respect to the sample interface the sum frequency light was s polarised, the 800 nm light s polarised and the infrared light p polarised, which is denoted as ssp polarisation in short. Spectra were acquired in the

3000-3550 cm^{-1} frequency region with two accumulations of 10 s, i.e., 20 s total measurement time. These short acquisition time allowed for measuring several flow on and flow off cycles for all pH solutions of interest between pH 3 and 11 before the sample got corroded (see Figure S8). The measured spectra were normalised for the frequency dependent infrared beam intensity using the non-resonant signal of MgO coated with a gold film. Generally, the detected absolute intensity varied between different samples and different measurement days despite the normalisation with the reference. To allow for a direct comparison of different measurements, we measured MgO in contact with a flowing 1 mM HCl (pH 3) solution during each series and used the maximum intensity of this spectrum to additionally normalise the data. Steady state spectra shown for example in Figure 1 were taken after sufficient equilibration time so that the signal intensity was constant and not changing anymore over time.

Phase-resolved sum frequency generation spectroscopy

The laser setup was constructed similar to a report in literature [42]. The laser source for the phase-resolved sum frequency generation spectroscopy was a Ti:sapphire amplifier (Astrella, Coherent) with a 1 kHz repetition rate, ca. 30 fs pulse duration and an output pulse energy of 7 mJ. The 800 nm output pulse was split into several parts. 2 mJ were used to generate the infrared beam for the experiments using an optical parametric amplifier (TOPAS PRIME, LIGHT CONVERSION) with a successive non-collinear difference frequency generation stage (NDFG, LIGHT CONVERSION). The resulting infrared beam had an energy of about 4 µJ at 3100 nm and a FWHM of about 380 cm^{-1}. The 800 nm beam was narrowed down by sending 1 mJ of the laser output through a pulse shaper, yielding pulses with a FWHM of about 20 cm^{-1} and an energy of 18 µJ. The narrowed 800 nm and infrared pulses were focused onto the local oscillator generator with a fused silica lens (LA4716-B, Thorlabs) and a CaF_2 lens (LA5042, Thorlabs), respectively. The local oscillator generator consisted of a 150 nm thick sputtered ZnO layer on top of a CaF_2 substrate (1 mm thickness, 25 mm diameter, Crystal GmbH). The 800 nm, infrared and local oscillator sum frequency pulses were collimated by a 60° off-axis parabolic mirror (MPD246-P01, Thorlabs). Afterwards, the local oscillator sum frequency light was passed through a 2 mm thick CaF_2 plate to induce a phase delay. All three beams were refocused onto the sample by a 60° off-axis parabolic mirror (MPD246-P01, Thorlabs). The incident angels for the 800 nm and infrared beam were ca. 60° and ca. 40° with respect to the sample surface normal, respectively. All measurements were done with s polarisation for the 800 nm and p polarisation for the infrared light. Both sum frequency pulses from the local oscillator generator and the sample were detected in s polarisation. The MgO substrate was mounted in a flow cell as described in the previous section. The reflected sample and local oscillator sum frequency light was collimated and refocused onto the slit off a spectrometer (Andor Shamrock 303i, Oxford Instruments) where the beams were dispersed by a grating and subsequently detected with a charge-coupled device image sensor (Andor Newton EMCCD, Oxford Instruments) at -75°C. The setup was purged with dry air for measurements of signals >3600 cm^{-1}. The obtained interference signals were processed as described in literature [43]. Briefly, the spectrum was inverse Fourier-transformed into the time domain, where one of the cross-terms was selected using a filter function. Next, the cross-term was Fourier-transformed back to the frequency domain. The same procedure was done for the gold coated MgO reference. Followingly, the processed sample spectrum was divided by the processed spectrum of the reference. Measurements of MgO in contact with D_2O (99.9%, Deutero GmbH) were used to correct for the different phase shift of the local oscillator sum frequency signal with the sample and reference sum frequency signals. Steady state spectra shown for example in Figure S3 were taken after sufficient equilibration time so that the signal intensity was constant and not changing anymore over time.

This setup was also used for conventional sum frequency generation spectroscopy for data shown in the supporting information. For this purpose, the local oscillator signal was blocked in between the off-axis parabolic mirrors. Different gratings of the spectrometer were used depending on the needed resolution or signal strength. In Section S3 a 1200 g/mm grating was used to compare the conventional and phase-resolved spectra. For the conventional sum frequency generation spectroscopy in Sections S5, S6 and S7 a grating with 600 g/mm was used.

Fitting of conventional sum frequency generation spectra

The normalised spectra were fitted using a real non-resonant contribution, $\chi_{NR}^{(2)}$, and complex Lorentzians for the resonant signal, $\chi_{R}^{(2)}$, as introduced in the literature [29]:

$$I_{SFG} \propto \left|\chi_{NR}^{(2)} \cdot e^{i\phi} + \chi_{R}^{(2)}\right|^2 \quad (4)$$

$$\text{with } \chi_{R}^{(2)}(\omega) = \sum_n \frac{A_n}{(\omega-\omega_n)+i\Gamma_\text{n}} \quad (5)$$

Phase-resolved sum frequency generation spectroscopy indicated that the non-resonant contribution was negligibly small, more details can be found in Section S3 of the SI. Therefore, we fitted all spectra with the non-resonant contribution set to 0, consequently also the phase ϕ was set to 0°. The spectra shown in the main text were fitted using two peaks for the resonant signal. The first peak around 3240 cm^{-1} showed a shift depending on the solution pH and whether the liquid was flowing or static. The second peak stayed constant for all solutions and both flowing and static liquid at a resonance frequency of ω_2=3437 cm^{-1} with a half width at half the maximum height (HWHM) of Γ_2= 92 cm^{-1}. The measurements depicted in Figure 1A with flowing liquid and different solution pH values were fitted with the first peak frequency $\omega_{1,flow}$ and both peak amplitudes $A_{1,flow}$ and $A_{2,flow}$ as free parameters. The linewidth of the first peak was fixed to a HWHM of $\Gamma_{1,\text{flow}}$=143 cm^{-1}, as it had no significant effect on the quality of the fits and keeping it constant enables a quantitative comparison of the amplitudes at different pH values [29]. The peak frequency $\omega_{1,flow}$ of the pH 10 and 11 solutions was fixed at the pH 9 value, because the peaks were too small to allow for stable fits. Measurements with static liquid, shown in Figure 1B, were fitted with a constant frequency and width of $\omega_{1,static}$=3268 cm^{-1} and $\Gamma_{1,\text{static}}$=57 cm^{-1}. The fits of the salt screening experiments from Figure 1C were done by only varying the amplitudes A_1 and A_2 of the peaks. The other values were constant at $\omega_{1,screening}$=3236 cm^{-1} with a HWHM of $\Gamma_{1,\text{screening}}$=143 cm^{-1} (the second peak stayed as before at ω_2=3437 cm^{-1} with a HWHM of Γ_2= 92 cm^{-1}). A complete summary of the fit parameters is given in Tables S1-S3. The fits of the combined phase-resolved and conventional sum frequency generation spectroscopy are discussed separately in Section S3.

Table S1. *Fit parameters of the spectra with flowing liquid shown in Figure 1A. The non-resonant signal and the associated phase are both set to 0. Peak frequencies* ω *and linewidths (HWHM)* Γ *are in [cm*$^{-1}$*], while the amplitude* A *is in [a.u.].*

	**pH 3**	**pH 3.3**	**pH 3.6**	**pH 4**	**pH 5**	**pH 5.5**	**pH 6**	**pH 7**	**pH 8**	**pH 9**	**pH 10**	**pH 11**
$\boldsymbol{A_{1,flow}}$	121	116	125	117	83	33	50	43	35	42	16	9
$\boldsymbol{\omega_{1,flow}}$	3240	3238	3231	3236	3242	3266	3253	3260	3267	3264	3264	3264
$\boldsymbol{\Gamma_{1,flow}}$							143					
$\boldsymbol{A_{2,flow}}$	46	50	46	49	38	36	26	23	28	24	24	24
$\boldsymbol{\omega_2}$							3437					
$\boldsymbol{\Gamma_2}$							92					

Table S2. *Fit parameters of the spectra with static liquid shown in Figure 1B. The non-resonant signal and the associated phase are both set to 0. Peak frequencies* ω *and linewidths (HWHM)* Γ *are in [cm*$^{-1}$*], while the amplitude* A *is in [a.u.].*

	**pH 3**	**pH 3.3**	**pH 3.6**	**pH 4**	**pH 5**	**pH 5.5**	**pH 6**	**pH 7**	**pH 8**	**pH 9**	**pH 10**	**pH 11**
$\boldsymbol{A_{1,static}}$	8	6	7	6	5	4	12	12	9	10	6	4
$\boldsymbol{\omega_{1,static}}$							3268					
$\boldsymbol{\Gamma_{1,static}}$							57					
$\boldsymbol{A_{2,static}}$	36	36	32	34	32	34	28	28	31	30	28	24
$\boldsymbol{\omega_2}$							3437					
$\boldsymbol{\Gamma_2}$							92					

Table S3. *Fit parameters of the spectra with flowing pH 3 solutions having ionic strengths ranging from 1-1000 mM shown in Figure 1C. The non-resonant signal and the associated phase are both set to 0. Peak frequencies* ω *and linewidths (HWHM)* Γ *are in [cm*$^{-1}$*], while the amplitude* A *is in [a.u.].*

	1 mM	***10 mM***	***100 mM***	***1000 mM***
$A_{1,flow}$	*126*	*72*	*50*	*33*
$\omega_{1,flow}$			*3236*	
$\Gamma_{1,flow}$			*143*	
$A_{2,flow}$	*37*	*29*	*23*	*24*
ω_2			*3437*	
Γ_2			*92*	

.

S2: Surface charge screening experiment at pH 11

Figure S2 depicts sum frequency generation spectroscopy measurements of MgO in contact with flowing pH 11 solutions with ionic strengths between 1-1000 mM. As in the main text, the spectra are normalised to the peak maximum of a pH 3 spectrum to allow for a better comparison. Contrary to measurements at pH 3 shown in Figure 1C, an increase of ionic strength has no influence on the signal intensity. This agrees with the hypothesis that the MgO surface is neutrally charged when in contact with a pH 11 solution, since then there is no net charge to be screened by the added salt. Furthermore, the additional Na^+ or Cl^- ions in the solution seem to have no observable secondary effect in our measurements, for example no net charging of the surface or water orientation by adsorption of Na^+ or Cl^- ions.

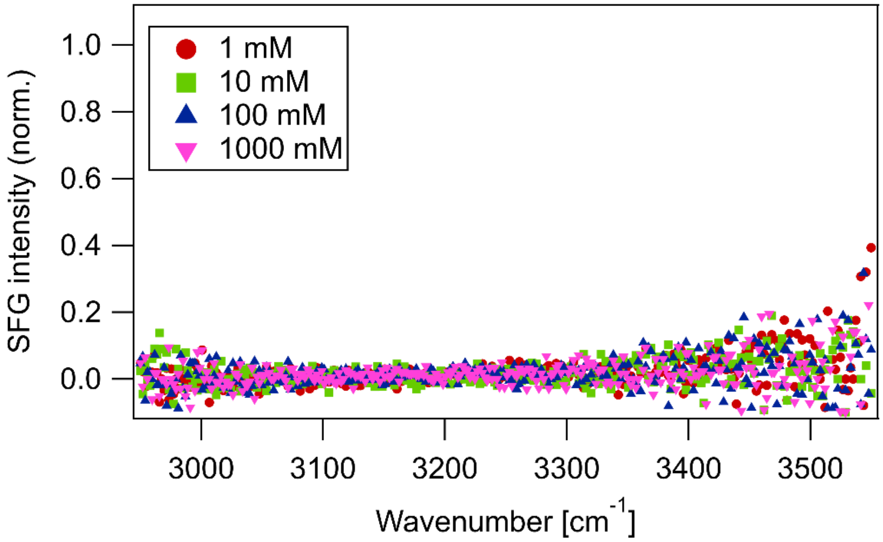

Figure S2. *Steady state sum frequency generation spectra of MgO(100) in contact with a flowing pH 11 solution. Solutions with ionic strengths from 1-1000 mM were measured by adding appropriate amounts of NaCl to a 1 mM NaOH solution. The spectra are normalised to the maximum intensity of a pH 3 solution in contact with MgO to allow for a better comparison to Figure 1C.*

S3: Conventional and phase-resolved sum frequency generation spectroscopy measurements using higher resolution

In conventional sum frequency generation spectroscopy the detected intensity (I_{SFG}) can be described by the electric fields of the incident visible (E_{vis}) and infrared (E_{IR}) light and the second-order nonlinear susceptibility ($\chi^{(2)}$) [29]:

$$I_{SFG} \propto |E_{vis}|^2 \cdot |E_{IR}|^2 \cdot \left|\chi^{(2)}\right|^2 \qquad (6)$$

$\chi^{(2)}$ is a complex quantity and the sign of the imaginary component reveals the orientation of interfacial species, e.g., if the hydrogens of a water molecule point towards or away from the surface [29]. Usually, the imaginary and real components of $\chi^{(2)}$ and their respective signs are inferred from peak fitting the detected intensity. However, the components can be measured directly by applying phase-resolved sum frequency generation spectroscopy [28], [44]. We performed conventional and phase-resolved sum frequency generation spectroscopy of MgO in contact with a flowing and a static 1 mM HCl (pH 3) solution. Compared to the data shown in the main text we measured over a broader frequency range with 5 min acquisition time and higher resolution which was not feasible for the kinetic measurements. The conventional sum frequency generation spectra depicted in Figure S3A were fitted with peaks having a positive amplitude, which gives a negative sign for the imaginary $\chi^{(2)}$, $Im(\chi^{(2)})$. Then, the fitted $Im(\chi^{(2)})$ agrees well with the measured $Im(\chi^{(2)})$ from phase-resolved measurements, which is shown in Figure S3B. In principle it was possible to fit the conventional sum frequency generation spectra well with and without a non-resonant contribution. As a non-resonant contribution close to 0 resulted in a significantly better agreement of the fitted real and imaginary $\chi^{(2)}$ with the measured real and imaginary $\chi^{(2)}$, we set the non-resonant signal to 0 throughout the fits of this study.

The assignment of the fitted peaks follows the argumentation from the main text (see Figure 1). MgO in contact with a flowing pH 3 solution featured an intense broad peak at 3237 cm^{-1} (HWHM Γ=167 cm^{-1}) from diffuse layer water, which is net oriented/polarised due to the MgO surface charge. This peak was shifted to 3262 cm^{-1} (Γ=104 cm^{-1}) for static liquid, which as discussed in the main text is likely due to weaker hydrogen bonding because of the decreased charge [39]. The higher resolution of the performed measurements revealed that the feature around 3440 cm^{-1} originates from two peaks, one centred at 3399 cm^{-1} (Γ=79 cm^{-1}) and one peak at 3474 cm^{-1} (Γ=41 cm^{-1}). We found that the intensity ratio of the 3399 cm^{-1} and 3474 cm^{-1} peaks seemed to depend on the MgO sample and the degree of dissolution the surface experienced. Unfortunately, no clear trend for the changes could be identified. Especially the peak at 3474 cm^{-1} was less pronounced in some measurements. In literature multiple peaks in this frequency region were assigned to adsorbed water on MgO at different surface sites and hydrogen bonding environments [7], [13], which is in agreement with our peak signs. The contribution from diffuse layer water in the 3400 cm^{-1} region [34], [36] is likely superimposed by the adsorbed water peaks and caused the intensity decrease of the adsorbed water peaks when the liquid flow was stopped. A significant contribution from the asymmetric stretch modes of water found at similar frequencies is unlikely, since it would have a different sign of $Im(\chi^{(2)})$ than the found symmetric stretch modes [45].

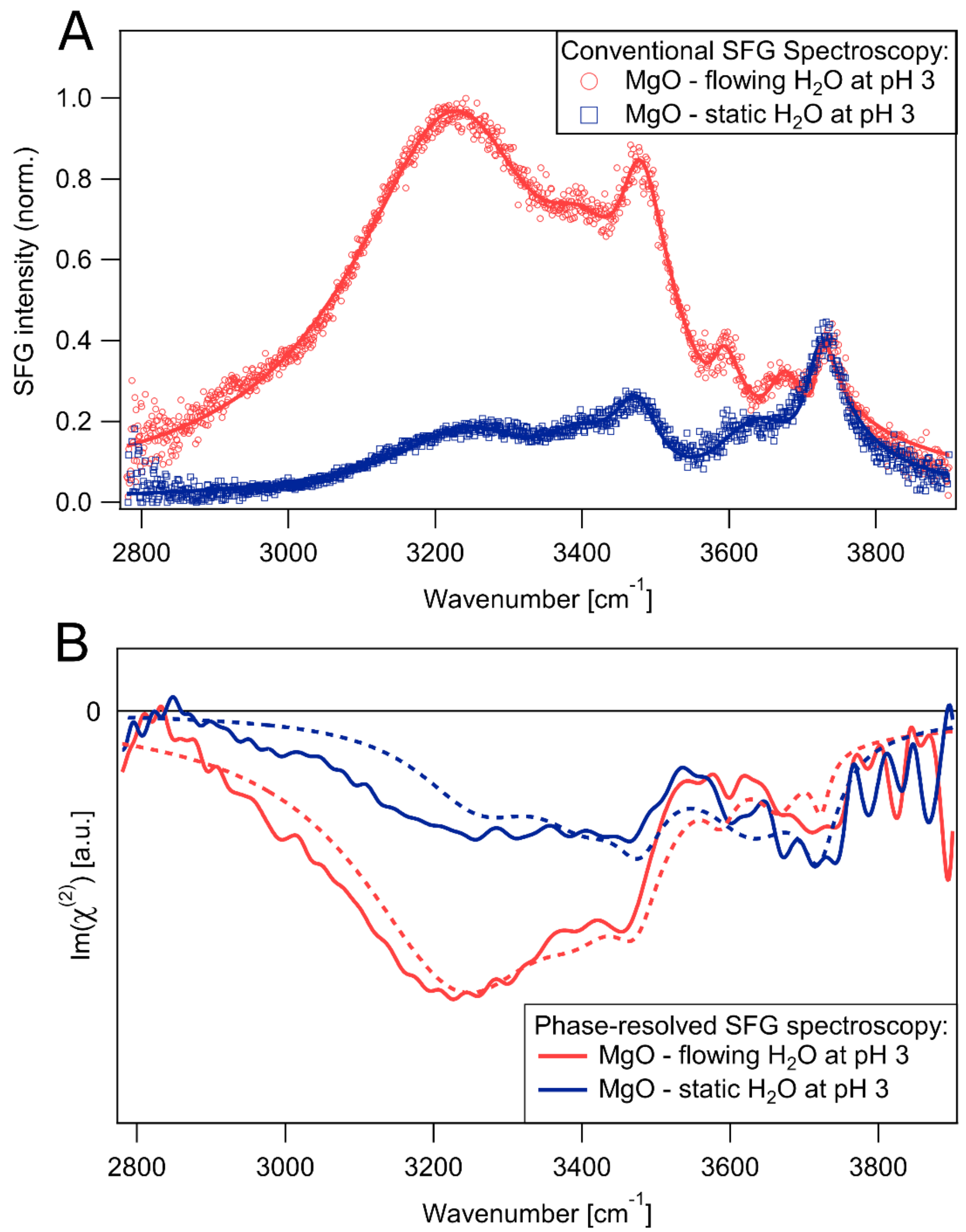

Figure S3. *Steady state sum frequency generation spectroscopy measurements of MgO(100) in contact with flowing and static 1 mM HCl (pH 3) solutions. A) Intensity (conventional) spectra, the solid lines represent fits to the data using complex Lorentzians. B) Phase-resolved spectra showing the measured imaginary component of* $\chi^{(2)}$*, alongside the calculated imaginary component from fitting in dashed lines.*

At high wavenumbers, close to the vibrations of gas phase water around 3700 cm^{-1}, the spectrum differs for flowing and static liquid. For static liquid two peaks were found at 3632 cm^{-1} (Γ=87 cm^{-1}) and 3720 cm^{-1} (Γ=30 cm^{-1}). The high frequency of these species is indicative of no to only weak hydrogen bonding and fits reported values for surface hydroxyls. In literature the peak at 3720 cm^{-1} was assigned to hydroxyls bound to Mg^{2+} on the surface, while the lower frequency peak at 3632 cm^{-1} was attributed to protonated surface oxygens [7], [8], [16], [38]. On the other hand, when flowing liquid was measured along the MgO crystal three moderately narrow peaks were found at 3587 cm^{-1} (Γ=23 cm^{-1}), 3667 cm^{-1} (Γ=34 cm^{-1}) and 3723 cm^{-1} (Γ=14 cm^{-1}). The peak at 3723 cm^{-1} matches again well with Mg^{2+} bound hydroxyls, whereas the

other two peaks do not fit previous literature reports. In a flowing pH 3 solution significant surface charging and dissolution is expected contrary to measurements with static liquid. Therefore, in theory the two peaks at 3587 cm^{-1} and 3667 cm^{-1} might belong to transient species occurring predominantly during dissolution, for example double protonated surface sites. It should be noted that transmission infrared spectra showed an absorption around 3540 cm^{-1} from the MgO substrates which in theory could modify the observed hydroxyl signals. Nevertheless, in Section S4 it is discussed that its influence on the spectra can be neglected.

Lastly, the orientation of the observed interfacial species can be derived from the fitting analysis and the phase-resolved measurements. All fitted peaks had a negative $Im(\chi^{(2)})$, which matches the phase-resolved spectra. Thus, all observed (symmetric) OH stretching vibrations are oriented similar with respect to the MgO surface. The observed surface hydroxyls must point with the hydrogen away from the surface, since they cannot penetrate the MgO crystal. Therefore, also all other observed species point with their hydrogen atoms on average away from the surface, as expected for a positively charged surface.

S4: Transmission infrared spectroscopy of the MgO sample

The signal obtained by sum frequency generation spectroscopy can be affected by absorptions from the sample, since both the infrared and 800 nm laser beams must pass through the sample before reaching the probed interface. Often solid samples contain water trapped within the crystal which usually gives absorption bands >3500 cm^{-1}. Figure S4A depicts transmission infrared spectroscopy (Tensor 37, Bruker) measurements of an exemplary MgO sample used within this article. Generally, the transmittance was well above 0.87 between 3000-4000 cm^{-1} and only little change was observable. A zoom into the OH-stretch region is shown in the inset of Figure S4A. Although the change in transmittance was arguably small, there was a decreasing trend observable towards 4000 cm^{-1}. Furthermore, an absorption peak was found around 3540 cm^{-1}, which most probably originated from a trapped OH-species like water. This weak absorption could potentially modulate the obtained sum frequency generation spectroscopy measurement in that region. However, contributions from the bulk of MgO substrates are usually cancelled out due to the normalisation of the spectrum with a gold coated MgO reference, which has identical properties to the MgO samples used in the measurements. Small fluctuations of the 3540 cm^{-1} absorption between different samples could still create artificial peaks, but the fact that the spectra between flowing and static liquid in Figure S3 varied significantly supports the assumptions that observed peaks >3500 cm^{-1} are from the interface, since the bulk MgO contribution is not affected whether the solution flows or not.

Alternatively, a possible influence from the MgO substrate absorption at ca. 3540 cm^{-1} on the sum frequency generation measurements can be probed by measuring MgO in contact with D_2O. The stretch vibrations of D_2O are located approximately between 2000-2800 cm^{-1}. Therefore, the spectrum should be flat in the O-H stretch region between 3000-3800 cm^{-1}. Figure S4B depicts a conventional sum frequency generation spectrum of MgO in contact with static D_2O. Only the onset of the MgO-OD peak is visible, which was found in literature to be centred around 2735 cm^{-1} [16]. The absence of any OH-stretch features agrees with our previous assumption that the MgO substrate absorption does not influence our interfacial experiments.

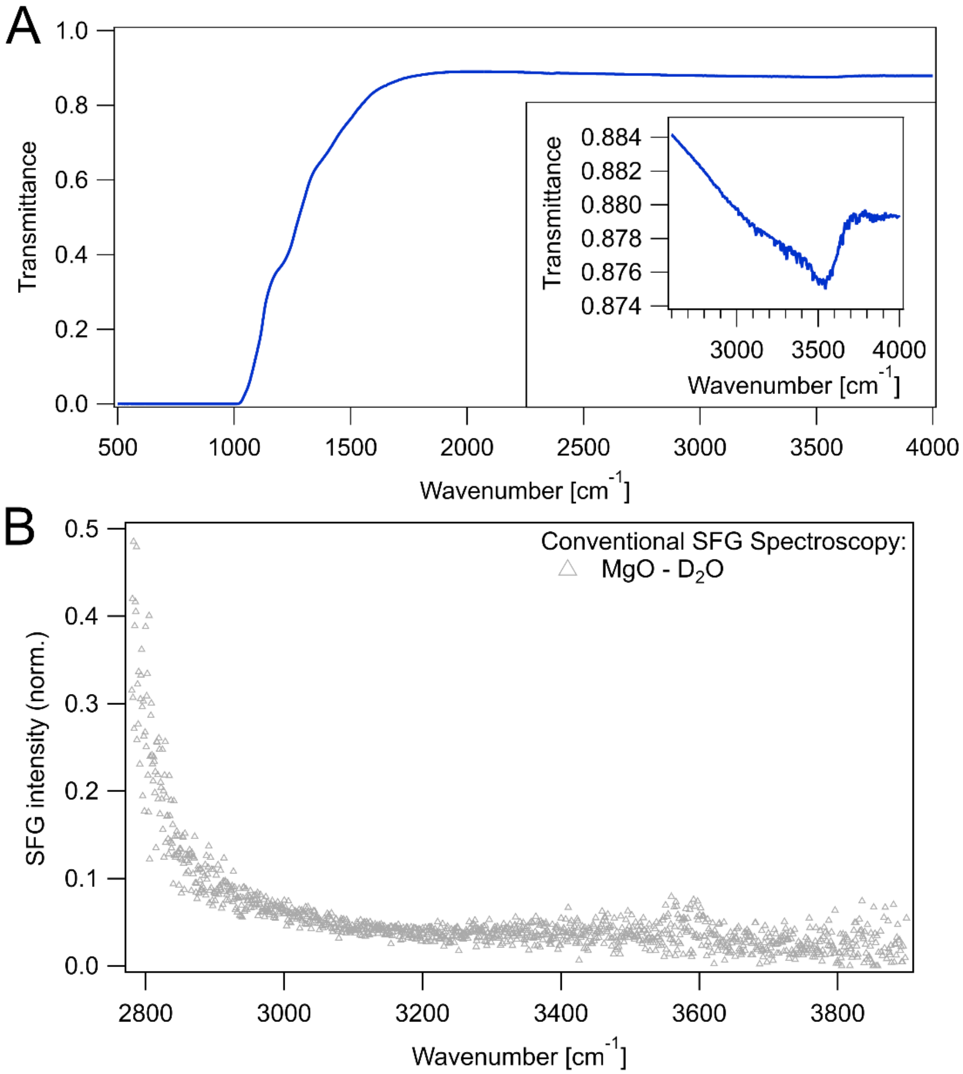

Figure S4. *A) Exemplary transmission infrared spectrum of one MgO sample with 2 mm thickness measured with a Bruker Tensor 37 spectrometer. The inset shows a magnified area of the OH-stretch region. The inset spectrum seems noisier around 3700 cm^{-1} due to fluctuating gas water absorption in the measurement chamber. B) Steady state conventional sum frequency generation spectrum of MgO in contact with static D_2O.*

S5: Probing the influence of the measurement cell on the flow on/off dynamics

The liquid in the flow cell is between 0.5-1 mm deep. In principle it is possible that the observed dynamics during the transition from flowing to static liquid could be affected by an artificial depletion of the H^+ concentration in the channel due to hindered diffusion form the solution reservoir. In case of flowing liquid, it is assumed that the solution exchange by the current is fast enough so that artificial concentration gradients in the cell are avoided. This can be roughly estimated by comparing the fluid velocity to the mean displacement of the solution ions x by Brownian motion in the time t, which can be calculated using the well-known equation [46]:

$$x = \sqrt{2 \cdot D \cdot t} \qquad (7)$$

The diffusion coefficients D for the included ions are $9.311 \cdot 10^{-5}$ cm²·s^{-1} for H^+, $0.706 \cdot 10^{-5}$ cm²·s^{-1} for Mg^{2+} and $2.032 \cdot 10^{-5}$ cm²·s^{-1} for Cl^- [47]. This yields a net diffusion of 0.14 mm·s^{-1} for H^+, 0.04 mm·s^{-1} for Mg^{2+} and 0.06 mm·s^{-1} for Cl^-. The fluid velocity for a volumetric flow of 6 mL·min^{-1} used throughout this article is 33 mm·s^{-1}, which is orders of magnitudes faster than the diffusion.

To investigate if there is an effect of the cell on the transition from flowing to static liquid, we performed sum frequency generation spectroscopy of MgO(100) in contact with a 1 mM HCl (pH 3) solution. Usually, all measurements were conducted at the centre of the cell above the channel. This time we varied the measurement position between the centre and the side of the cell, as sketched in Figure S5B. At the side of the cell the liquid layer is only 0.5 mm deep and the exchange of the fluid is assumed to be significantly worse than at the channel. Figure S5A shows the integrated signal intensity of continuously measured 20 s spectra. The liquid was flowing during the first 10 measurements and stopped afterwards for the remaining 40 spectra. It is evident that there was quite some fluctuation in the intensity decay after the fluid flow was stopped. It took around 400 s on average to reach a steady state. However, there were outliers with shorter and longer duration and there was also no consistent trend between channel and side measurements. Theoretically on the side there should be less H^+ supply from the bulk/reservoir due to a lower liquid depth beneath the surface and worse fluid exchange, even when the liquid is flowing. Furthermore, it is unlikely that the strong fluctuations of the intensity decay after switching off the liquid flow were caused by the cell. Such fluctuations were also observed for other kinetic experiments measured solely at the channel and seem to occur when not exactly the same spot on the MgO substrate was probed throughout the whole series. Thus, it is assumed that fluctuations of the MgO surface properties overshadow any possible effects from the measurement cell. This is also the reason for the data spread in Figure 3 and why we collected a substantial number of measurements to get a trustworthy estimate of the reaction order. In Section S8 this is further discussed based on the observed anisotropic dissolution of the MgO surface.

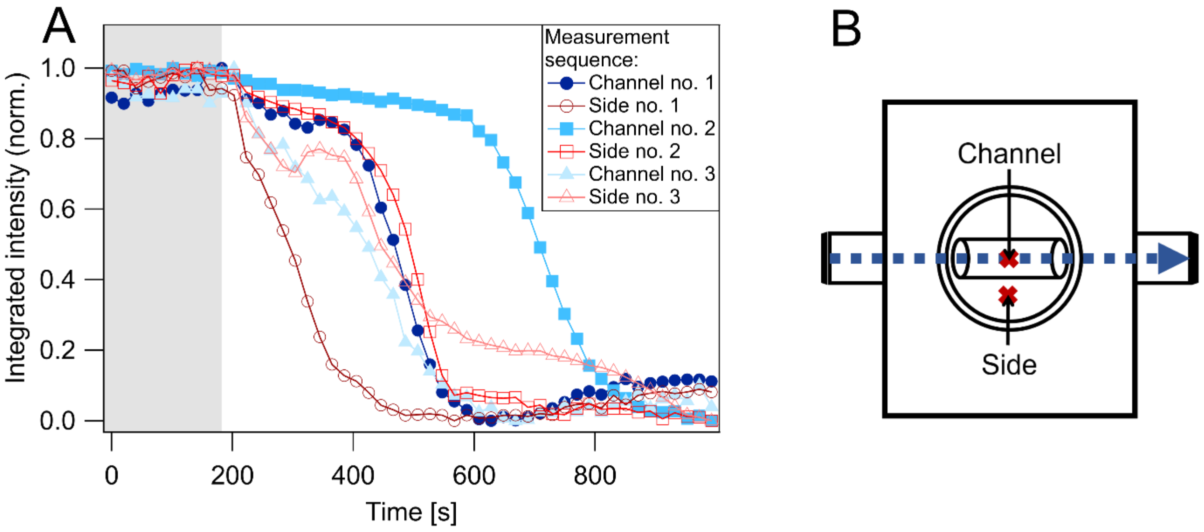

Figure S5. *A) Sum frequency generation spectroscopy of MgO(100) in contact with a 1 mM HCl solution where the measurement cell was moved to shift the beam overlap between the channel and the side of the cell. Continuous 20 s spectra were recorded and the integrated intensity is plotted versus the measurement time. The liquid was flowing in the grey area, afterwards the solution was kept static. The data is scaled to lie between 0 to 1 for a better comparability of the intensity decay. The data points are connected by a line to guide the eye. B) Drawings of the flow cell with the beam overlap positions for the channel and side (off-channel) measurements marked. The blue arrow indicates the flow of the liquid.*

S6: The effect of different fluid flow rates on the observed spectrum

Throughout the main text a fluid flow rate of 6 mL·min^{-1} was used. To test if the flow rate influences the experiments, we measured sum frequency generation spectroscopy of MgO(100) in contact with pH 3, 5 and 5.5 solutions with an ionic strength of 1 mM and varied the flow rates, as shown in Figure S6. For a better comparability the spectra were normalised to the maximum amplitude of the MgO-pH 3 spectrum with 6 mL·min^{-1} flow.

No effect of the flow rate on the peak intensities was observed with a liquid phase at pH 3 - even at very high flow rates around 270 mL·min^{-1}, as shown in Figure S6A. The invariance of the measured intensity at pH 3 to the flow rate supports the argumentation from the main text that the surface charge is the main cause for the intensity increase under flowing liquid. It was discussed that below pH 4 the surface is saturated with adsorbed H^+. Consequently, an increase of the flow rate at pH 3 was not able to further charge the MgO surface. Followingly, a direct influence of the liquid motion on the molecular water orientation was found to be negligible.

For a liquid phase with pH 5 and 5.5, depicted in Figure S6B, C, no significant intensity increase was detected at moderate laminar flow rates until 13 mL·min^{-1} as well. Contrary, a moderate intensity increase was observed when the flow speed was elevated to about 150 mL·min^{-1}, where a breakdown of the laminar flow is expected. The reason for the increase is yet to be fully understood. It could be that the induced turbulent flow affects the boundary layer and enhanced mixing increases the availability of interfacial H^+, which in turn can lead to an increase of the surface charge. Theoretically, there should be also a flow speed at which the transition from flowing to static liquid starts to blur, unfortunately we could not achieve low and stable enough flow rates with our equipment.

In conclusion, the influence of the flow rate can be neglected at moderate laminar flow speeds used within this article, just high (turbulent) flow seems to influence the surface charge for pH solutions outside of the charge saturation regime.

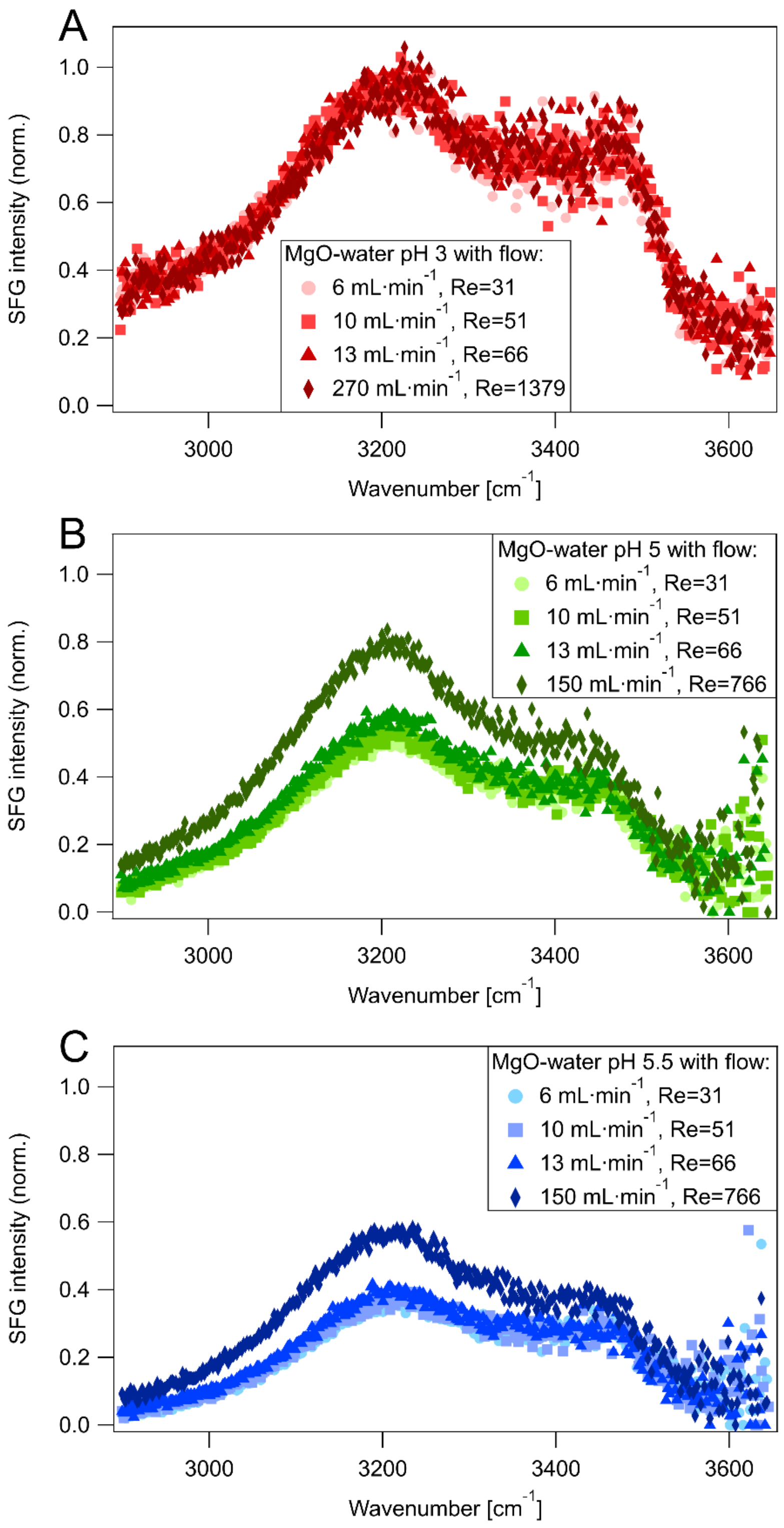

Figure S6. *Steady state sum frequency generation spectroscopy measurements of MgO(100) in contact with A) pH 3, B) pH 5 and C) pH 5.5 solutions with 1 mM ionic strength. During the measurements the volumetric flow of the liquid was increased and after > 2 min equilibration time spectra were recorded. Additionally, the Reynolds number (Re) for the respective volumetric flow is given.*

S7: Effect of Mg^{2+} addition on the signal intensity and flow on/off transition

The dissolution reaction of MgO under acidic conditions consumes in total two H^+ and releases Mg^{2+} and H_2O into the liquid phase. Mg^{2+} in the interfacial region could slow down the net dissolution reaction based on the chemical equilibrium and a possible backreaction, which in turn should affect the observed decrease of the signal intensity during the transition from flowing to static liquid shown in Figure 2A. To investigate the possible influence of Mg^{2+} we measured sum frequency generation spectra of MgO in contact with 1 mM HCl (pH 3) solutions with 1, 10 and 100 mM $MgCl_2$ added. To rule out that the dissolution dynamics or signal intensities of the MgO sample change during the experiment, for example due to Mg^{2+} precipitation, we measured before and after every $MgCl_2$ solution a 1 mM HCl (pH 3) solution without additional salt. Figure S7A depicts the integrated signal intensity of 20 s spectra measured back-to-back as a function of time. The liquid was flowing in the first 10 measurements and stopped afterwards for the remaining 40 spectra. Exemplary spectra with flowing liquid are shown in Figure S7B. For better comparability they are normalised to the peak maximum of the first pH 3 measurement. The test measurements with 1 mM HCl solutions were within experimental fluctuations constant, both in terms of the intensity decrease upon switching off the flow (Figure S7A) and spectral shape (Figure S7B). Therefore, we conclude that the MgO surface maintained consistent dissolution behaviour and surface charge characteristics throughout the experiment and no drift due to the Mg^{2+} salts was observed.

Upon addition of 1 mM $MgCl_2$ to the measurement solution no significant difference to measuring pure 1 mM HCl was observed. Neither the intensity decrease after stopping the flow (Figure S7A) nor the overall spectral shape (Figure S7B) showed significant deviations. In contrast, higher $MgCl_2$ concentrations clearly affected the measurements. Two major effects were observed. First, the overall intensity in Figure S7B decreased by around 40% for 10 mM $MgCl_2$ and 60% for 100 mM $MgCl_2$ addition compared the pure 1 mM HCl. This intensity decrease can be explained by surface charge screening effects as discussed in the main text in Figure 1C. Second, the relative intensity decrease after liquid flow was switched off, shown in Figure S7A, was faster by around 20% for 10 mM $MgCl_2$ and around 50% for 100 mM $MgCl_2$ compared to 1 mM $MgCl_2$ addition or pure 1 mM HCl. Thus, Mg^{2+} seems to indeed influence the dissolution/discharging dynamics of the MgO surface. In literature it was found that solvated Mg^{2+} ions strongly affect the water reorientation and dynamics in its hydration shell [48], which should also affect the diffusion of H^+ at high Mg^{2+} concentrations. As discussed in the main text in Figure 2B, the charging reaction of the MgO surface by H^+ adsorption is primarily limited by H^+ diffusion under static liquid conditions. A faster discharging of the MgO surface in contact with static liquid would be expected if the H^+ diffusion is slowed down by high concentrations of Mg^{2+}, which matches our experimental observations. However, the lack of any direct effect of Mg^{2+} on the chemical equilibrium of the surface reaction is surprising to us and opposite to observations for other materials such as CaF_2 [27].

In summary, it was found that Mg^{2+} can influence measurements of the MgO interfacial structure and dissolution. However, in the limit of low Mg^{2+} concentrations there was no effect observed, which is suggested by the similarity of our measurements using 1 mM HCl and 1 mM HCl + 1 mM $MgCl_2$ solutions. Followingly, we assume that within this study all experiments are done in the limit where there is no significant influence by Mg^{2+} yet.

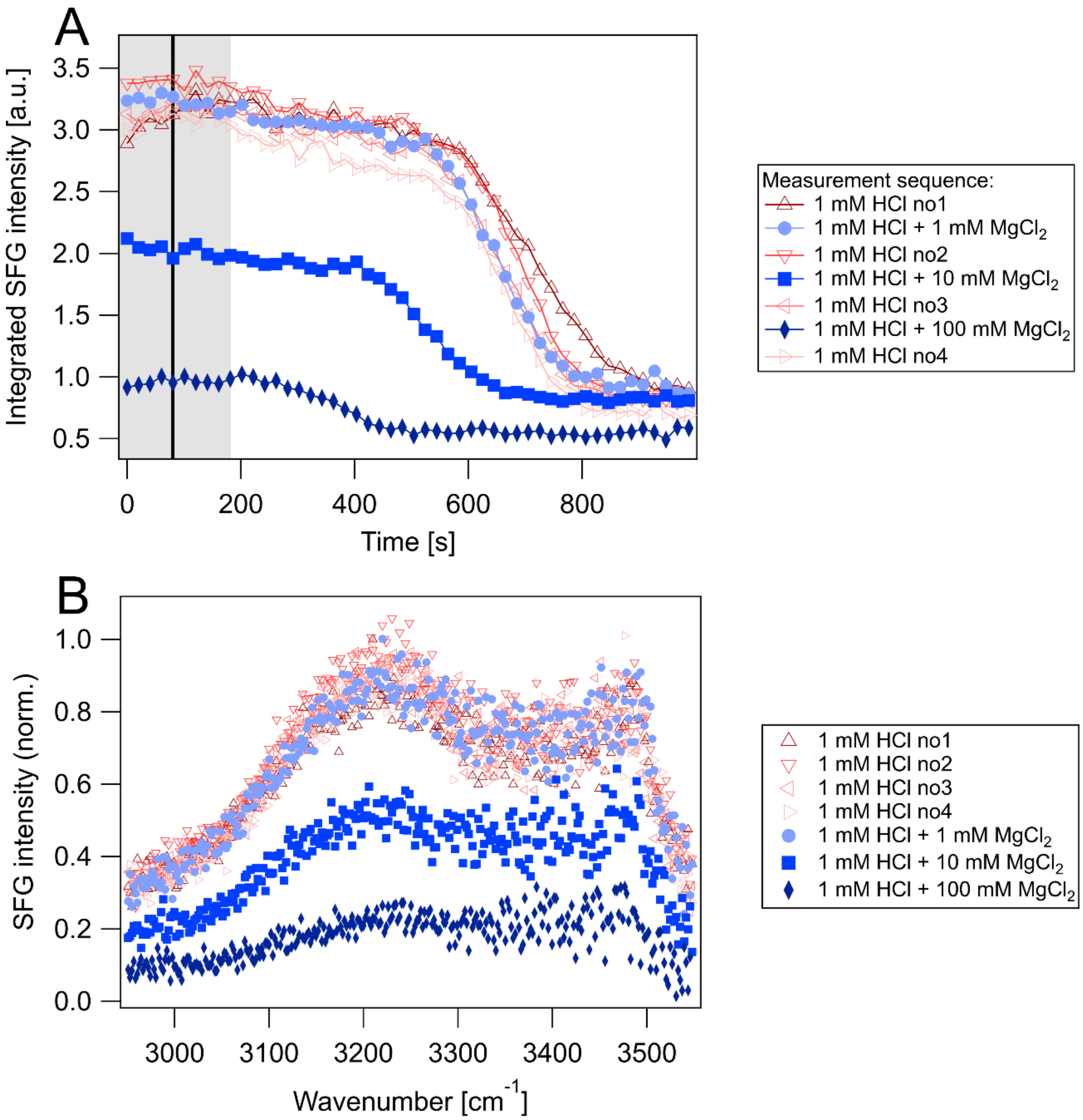

Figure S7. *Sum frequency generation spectroscopy measurements of MgO(100) in contact with 1 mM HCl (pH 3) solutions containing 0-100 mM* $MgCl_2$*. A) Integrated intensity of 20 s spectra from continuous measurements. The liquid was flowing in the grey shaded area, afterwards the solution was kept static. Exemplary steady state spectra, shown in B), are taken from the time around 100 s indicated by the vertical black line. The spectra in B) are normalised to the maximum intensity of the first 1 mM HCl measurement.*

<u>S8: Effect of dissolution on the MgO surface</u>

The effect of the dissolution reaction on the MgO(100) surface was monitored with the optical microscopy unit of a confocal Raman imaging microscope (WITec alpha 300 ARS). Microscopy images with 100x magnification (EC Epiplan-Neofluar 100x/0.9 DIC lens, Carl Zeiss AG) are presented in Figure S8. Figure S8A shows the pristine MgO(100) surface before any measurements or sample treatment. The surface appears smooth and featureless. After the substrate was brought into contact with acidic pH solutions holes in the low µm to sub-µm range were forming on the surface, as shown in Figure S8B. This image was taken after the MgO substrate was used in a measurement series equivalent to the data shown in Figure 2A. The observed formation of holes is consistent with literature reports using atomic force microscopy [18]. The hole formation became more evident, as depicted in Figure S8C, when the MgO sample was used for another series. Interestingly, it is observable that the whole formation across the surface was not isotropic, but rather smaller patches with high amounts of holes formed which were interrupted by areas with less dissolution marks.

The anisotropic dissolution of the surface might explain the variability of the observed dissolution curves at different measurement spots observed for example in Figure S5. We assume that the dissolution rate is slightly different at already strongly dissolved parts of the surface due to newly formed kink sites and edges on the crystal surface. The diameter of the area probed by our laser beams is around 100 µm, which means that depending on the spot position the detected average surface dissolution may differ if a more or less dissolved part of the sample is measured. Organic residues, which are visible in the microscopy pictures, e.g., Figure S8B, were effectively removed by heating the samples prior to sum frequency generation experiments, which can be seen by the lack of aliphatic C-H stretch vibrations between 2800-3000 cm^{-1} in the spectra from Figure S3 and S4B.

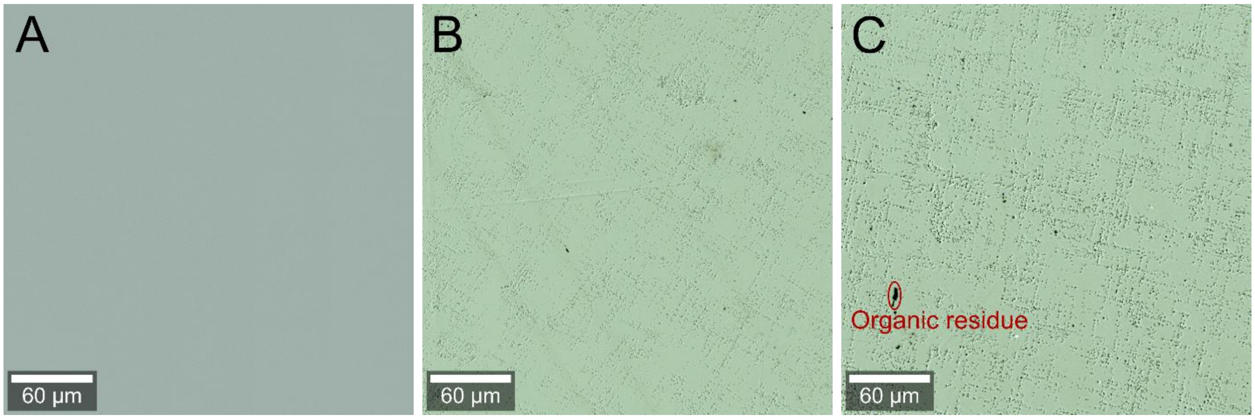

Figure S8. *Exemplary microscopy images of a MgO substrate with 100x magnification. All pictures have the same contrast settings. A) Pristine surface before any measurements. B) After one measurement series equivalent to Figure 2A. C) After two measurement series. The sample was not heated before microscopy, thus occasional some organic residues in dark black are visible on the surface in B) and C), such as the one marked in red in C).*

References

[1] J. C. Védrine, "Metal Oxides in Heterogeneous Oxidation Catalysis: State of the Art and Challenges for a More Sustainable World," *ChemSusChem*, vol. 12, no. 3, pp. 577–588, 2019, doi: 10.1002/cssc.201802248.

[2] J. Zhang, "Recent advance of MgO expansive agent in cement and concrete," *J. Build. Eng.*, vol. 45, no. September 2021, p. 103633, 2022, doi: 10.1016/j.jobe.2021.103633.

[3] E. Comini *et al.*, "Metal oxide nanoscience and nanotechnology for chemical sensors," *Sensors Actuators, B Chem.*, vol. 179, pp. 3–20, 2013, doi: 10.1016/j.snb.2012.10.027.

[4] J. L. Bañuelos *et al.*, "Oxide- and Silicate-Water Interfaces and Their Roles in Technology and the Environment," *Chemical Reviews*, vol. 123, no. 10. pp. 6413–6544, 2023, doi: 10.1021/acs.chemrev.2c00130.

[5] J. Balajka, U. Aschauer, S. F. L. Mertens, A. Selloni, M. Schmid, and U. Diebold, "Surface Structure of TiO2 Rutile (011) Exposed to Liquid Water," *J. Phys. Chem. C*, vol. 121, no. 47, pp. 26424–26431, 2017, doi: 10.1021/acs.jpcc.7b09674.

[6] C. A. Ohlin, E. M. Villa, J. R. Rustad, and W. H. Casey, "Dissolution of insulating oxide materials at the molecular scale," *Nat. Mater.*, vol. 9, no. 1, pp. 11–19, 2010, doi: 10.1038/nmat2585.

[7] P. J. Anderson, R. F. Horlock, and J. F. Oliver, "Interaction of water with the magnesium oxide surface," *Trans. Faraday Soc.*, vol. 61, no. 0, pp. 2754–2762, Jan. 1965, doi: 10.1039/TF9656102754.

[8] N. M. Adhikari, A. Tuladhar, Z. Wang, J. J. De Yoreo, and K. M. Rosso, "No Hydrogen Bonding between Water and Hydrophilic Single Crystal MgO Surfaces?," *J. Phys. Chem. C*, vol. 125, no. 47, pp. 26132–26138, 2021, doi: 10.1021/acs.jpcc.1c06486.

[9] M. Foster, M. Furse, and D. Passno, "An FTIR study of water thin films on magnesium oxide," *Surf. Sci.*, vol. 502–503, pp. 102–108, 2002, doi: 10.1016/S0039-6028(01)01906-9.

[10] J. T. Newberg *et al.*, "Autocatalytic surface hydroxylation of MgO(100) terrace sites observed under ambient conditions," *J. Phys. Chem. C*, vol. 115, no. 26, pp. 12864–12872, 2011, doi: 10.1021/jp200235v.

[11] M. I. McCarthy, G. K. Schenter, C. A. Scamehorn, and J. B. Nicholas, "Structure and Dynamics of the Water/MgO Interface," *J. Phys. Chem.*, vol. 100, no. 42, pp. 16989–16995, 1996, doi: 10.1021/JP961373I.

[12] M. Sassi and K. M. Rosso, "First principles simulations of MgO(100) surface hydration at ambient conditions," *Phys. Chem. Chem. Phys.*, vol. 26, no. 3, pp. 2269–2276, 2023, doi: 10.1039/d3cp04848a.

[13] M. Odelius, "Mixed molecular and dissociative water adsorption on MgO[100]," *Phys. Rev. Lett.*, vol. 82, no. 19, pp. 3919–3922, 1999, doi: 10.1103/PhysRevLett.82.3919.

[14] P. Thissen, V. Thissen, S. Wippermann, Y. J. Chabal, G. Grundmeier, and W. G. Schmidt, "PH-dependent structure and energetics of H2O/MgO(100)," *Surf. Sci.*, vol. 606, no. 11–12, pp. 902–907, 2012, doi: 10.1016/j.susc.2012.01.018.

[15] G. Di Liberto, F. Maleki, and G. Pacchioni, “pH Dependence of MgO, TiO2 and γ-Al2O3 Surface Chemistry from First Principles,” *J. Phys. Chem. C*, vol. 126, pp. 10216–10223, 2022, doi: https://doi.org/10.1021/acs.jpcc.2c02289.

[16] N. M. Adhikari, P. Zarzycki, Z. Wang, and K. M. Rosso, “pH-dependent reactivity of water at MgO(100) and MgO(111) surfaces,” *Phys. Chem. Chem. Phys.*, no. 27, pp. 4343–4354, 2025, doi: 10.1039/d4cp04223a.

[17] M. Robinson, J. A. Pask, and D. W. Fuerstenau, “Surface Charge of Alumina and Magnesia in Aqueous Media,” *J. Am. Ceram. Soc.*, vol. 47, no. 10, pp. 516–520, Oct. 1964, doi: 10.1111/J.1151-2916.1964.TB13801.X.

[18] M. F. Suárez and R. G. Compton, “Dissolution of magnesium oxide in aqueous acid: An atomic force microscopy study,” *J. Phys. Chem. B*, vol. 102, no. 37, pp. 7156–7162, 1998, doi: 10.1021/jp982260x.

[19] R. L. Segall, R. S. C. Smart, and P. S. Turner, “Ionic oxides: Distinction between mechanisms and surface roughening effects in the dissolution of magnesium oxide,” *J. Chem. Soc. Faraday Trans. 1 Phys. Chem. Condens. Phases*, vol. 74, pp. 2907–2912, 1978, doi: 10.1039/F19787402907.

[20] O. Fruhwirth, G. W. Herzog, I. Hollerer, and A. Rachetti, “Dissolution and hydration kinetics of MgO,” *Surf. Technol.*, vol. 24, no. 3, pp. 301–317, Mar. 1985, doi: 10.1016/0376-4583(85)90080-9.

[21] P. Raschman and A. Fedoročková, “Dissolution kinetics of periclase in dilute hydrochloric acid,” *Chem. Eng. Sci.*, vol. 63, no. 3, pp. 576–586, 2008, doi: 10.1016/j.ces.2007.10.004.

[22] A. Fedoročková and P. Raschman, “Effects of pH and acid anions on the dissolution kinetics of MgO,” *Chem. Eng. J.*, vol. 143, no. 1–3, pp. 265–272, 2008, doi: 10.1016/j.cej.2008.04.029.

[23] D. A. Vermilyea, “The Dissolution of MgO and Mg(OH)2 in Aqueous Solutions,” *J. Electrochem. Soc.*, vol. 116, no. 9, p. 1179, 1969, doi: 10.1149/1.2412273.

[24] D. Lis, E. H. G. Backus, J. Hunger, S. H. Parekh, and M. Bonn, “Liquid flow along a solid surface reversibly alters interfacial chemistry,” *Science (80-.).*, vol. 344, no. 6188, pp. 1138–1142, 2014, doi: 10.1126/science.1253793.

[25] J. Schaefer, E. H. G. Backus, and M. Bonn, “Evidence for auto-catalytic mineral dissolution from surface-specific vibrational spectroscopy,” *Nat. Commun.*, vol. 9, no. 1, pp. 1–6, 2018, doi: 10.1038/s41467-018-05762-9.

[26] P. Ober, W. Q. Boon, M. Dijkstra, E. H. G. Backus, R. van Roij, and M. Bonn, “Liquid flow reversibly creates a macroscopic surface charge gradient,” *Nat. Commun.*, vol. 12, no. 1, pp. 1–11, 2021, doi: 10.1038/s41467-021-24270-x.

[27] P. Ober, J. Hunger, S. H. Kolbinger, E. H. G. Backus, and M. Bonn, “Ion Adsorption and Desorption at the CaF2-Water Interface Probed by Flow Experiments and Vibrational Spectroscopy,” *Angewandte Chemie - International Edition*, vol. 61, no. 46. 2022, doi: 10.1002/anie.202207017.

[28] Y. R. Shen, “Surface properties probed by second-harmonic and sum-frequency generation,” *Nature*, vol. 337, no. 9, p. 519, 1989, [Online]. Available: https://www.nature.com/articles/337519a0.pdf.

[29] A. G. Lambert, P. B. Davies, and D. J. Neivandt, “Implementing the theory of sum frequency generation vibrational spectroscopy: A tutorial review,” *Appl. Spectrosc. Rev.*, vol. 40, no. 2, pp. 103–145, 2005, doi: 10.1081/ASR-200038326.

[30] K. C. Jena, P. A. Covert, and D. K. Hore, “The effect of salt on the water structure at a charged solid surface: Differentiating second- and third-order nonlinear contributions,” *J. Phys. Chem. Lett.*, vol. 2, no. 9, pp. 1056–1061, 2011, doi: 10.1021/jz200251h.

[31] S. Ong, X. Zhao, and K. B. Eisenthal, “Polarization of water molecules at a charged interface: second harmonic studies of the silica/water interface,” *Chem. Phys. Lett.*, vol. 191, no. 3–4, pp. 327–335, 1992, doi: 10.1016/0009-2614(92)85309-X.

[32] K. Bourikas, J. Vakros, C. Kordulis, and A. Lycourghiotis, “Potentiometric mass titrations: Experimental and theoretical establishment of a new technique for determining the point of zero charge (PZC) of metal (hydr)oxides,” *J. Phys. Chem. B*, vol. 107, no. 35, pp. 9441–9451, 2003, doi: 10.1021/jp035123v.

[33] S. Sun, J. Schaefer, E. H. G. Backus, and M. Bonn, “How surface-specific is 2nd-order non-linear spectroscopy?,” *J. Chem. Phys.*, vol. 151, no. 23, 2019, doi: 10.1063/1.5129108.

[34] B. Rehl *et al.*, “Water Structure in the Electrical Double Layer and the Contributions to the Total Interfacial Potential at Different Surface Charge Densities,” *J. Am. Chem. Soc.*, vol. 144, no. 36, pp. 16338–16349, 2022, doi: 10.1021/jacs.2c01830.

[35] K. A. Becraft and G. L. Richmond, “In situ vibrational spectroscopic studies of the CaF2/H2O interface,” *Langmuir*, vol. 17, no. 25, pp. 7721–7724, 2001, doi: 10.1021/la011133g.

[36] Y. C. Wen *et al.*, “Unveiling Microscopic Structures of Charged Water Interfaces by Surface-Specific Vibrational Spectroscopy,” *Phys. Rev. Lett.*, vol. 116, no. 1, 2016, doi: 10.1103/PhysRevLett.116.016101.

[37] M. Sovago, R. Kramer Campen, H. J. Bakker, and M. Bonn, “Hydrogen bonding strength of interfacial water determined with surface sum-frequency generation,” *Chem. Phys. Lett.*, vol. 470, no. 1–3, pp. 7–12, 2009, doi: 10.1016/j.cplett.2009.01.009.

[38] E. Carrasco *et al.*, “Thickness-dependent hydroxylation of MgO(001) thin films,” *J. Phys. Chem. C*, vol. 114, no. 42, pp. 18207–18214, 2010, doi: 10.1021/jp105294e.

[39] D. E. Gragson and G. L. Richmond, “Investigations of the Structure and Hydrogen Bonding of Water Molecules at Liquid Surfaces by Vibrational Sum Frequency Spectroscopy,” *J. Phys. Chem. B*, vol. 102, no. 20, 1998, doi: https://doi.org/10.1021/jp9806011.

[40] F. M. White, *Fluid Mechanics*, SEVENTH ED. McGraw-Hill, 2011.

[41] J. Kestin, M. Sokolov, and W. A. Wakeham, “Viscosity of liquid water in the range minus 8 degrees to 150 degrees,” *J. Phys. Chem. Ref. Data*, vol. 7, pp. 941–948, 1978, doi: https://doi.org/10.1063/1.555581.

[42] H. Vanselous and P. B. Petersen, “Extending the Capabilities of Heterodyne-Detected Sum-Frequency Generation Spectroscopy: Probing Any Interface in Any Polarization Combination,” *J. Phys. Chem. C*, vol. 120, no. 15, pp. 8175–8184, 2016, doi: 10.1021/acs.jpcc.6b01252.

[43] S. Nihonyanagi, S. Yamaguchi, and T. Tahara, “Direct evidence for orientational flip-flop of water

molecules at charged interfaces: A heterodyne-detected vibrational sum frequency generation study," *J. Chem. Phys.*, vol. 130, no. 20, 2009, doi: 10.1063/1.3135147.

[44] Y. R. Shen, "Phase-sensitive sum-frequency spectroscopy," *Annu. Rev. Phys. Chem.*, vol. 64, pp. 129–150, 2013, doi: 10.1146/annurev-physchem-040412-110110.

[45] M. Buessler, S. Maruyama, M. Zelenka, H. Onishi, and E. H. G. Backus, "Unravelling the interfacial water structure at the photocatalyst strontium titanate by sum frequency generation spectroscopy," *Phys. Chem. Chem. Phys.*, vol. 25, no. 45, pp. 31471–31480, 2023, doi: 10.1039/d3cp03829g.

[46] H. C. Berg, "Diffusion: Microscopic Theory," in *Random Walks in Biology*, Princeton University Press, 1993, p. 10.

[47] D. R. Lide, Ed., *CRC handbook of chemistry and physics. Vol. 85*. CRC press, 2004.

[48] W. Li and Z. Sun, "Second Hydration Shell of Mg2+: Competition between Ion-Water Interaction and Hydrogen Bonding Interaction," *J. Phys. Chem. Lett.*, pp. 12673–12679, 2024, doi: 10.1021/acs.jpclett.4c02771.
